# Electricity-Induced Simultaneous *in Situ* Remediation of Arsenic and Polycyclic Aromatic Hydrocarbons in Groundwater at a Former Wood Treatment Site – a Field Pilot Study

**DOI:** 10.1021/acsestwater.6c00318

**Published:** 2026-06-02

**Authors:** Jurate Kumpiene, Ivan Carabante, Erkki Lindberg, Spencer Long, Nicola Pratt, Phyllis Lam, Andrew B. Cundy

**Affiliations:** † Waste Science and Technology, Luleå University of Technology, 97187 Luleå, Sweden; ‡ Ekogrid Oy, Nuijamiestentie 7, 00400 Helsinki, Finland; § School of Ocean and Earth Science, University of Southampton, National Oceanography Centre Southampton, European Way, Southampton SO14 3ZH, Hampshire, U.K.

**Keywords:** creosote, wood impregnation, contaminated soil, microbial and fungal community structure, stabilization

## Abstract

Remediation of former wood treatment sites is challenging due to the presence of contaminants with distinct physicochemical properties, such as arsenic (As) and polycyclic aromatic hydrocarbons (PAHs). This study evaluated a low-voltage electricity-induced soil remediation method designed to immobilize As while simultaneously degrading PAH *in situ*. A field pilot experiment was conducted at a highly contaminated site using iron (Fe) electrodes supplying pulsed direct current to promote PAH oxidation and Fe release from electrodes for As immobilization. Groundwater in five wells was monitored for concentrations of contaminants, their degradation byproducts, and microbial and fungal community structures. Over two years, dissolved PAH16 concentrations decreased by 62–94% across wells, with no accumulation of oxygenated or nitrogen-containing PAH. Dissolved As concentrations declined by up to 88% at low PAH levels, but reductions were weaker (55–57%) and more variable at very high PAH concentrations (hundreds to thousands μg L^–1^). Microbial communities, both prokaryotic and fungal, were characterized by taxa often found in contaminated aquifers and soils, with enrichment of PAH-degrading and As-tolerant *Pseudomonas*, *Rugosibacter*, and *Duganella*, but showed no adverse effect of the treatment. Overall, the method promoted concurrent PAH degradation and As immobilization with minimal secondary impacts, demonstrating potential for remediation of mixed-pollutant soils.

## Graphical abstract

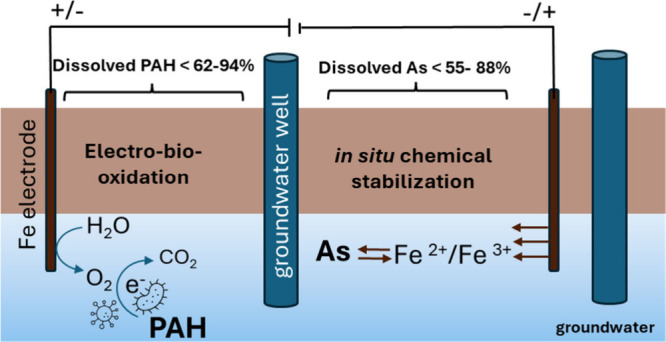

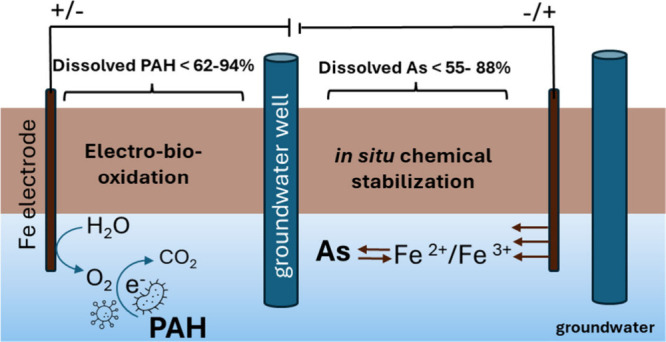

## 1 Introduction

Remediation of soil contaminated with complex mixtures of contaminants poses significant challenges, particularly in selecting appropriate technologies capable of managing multiple substances simultaneously. *In situ* biological methods are generally preferred due to their environmental benefits and potential to preserve soil health when degrading organic pollutants. However, their effectiveness can be severely limited by the presence of high concentrations of potentially toxic trace elements (TEs). In such cases, toxicity caused by TEs can be mitigated either by removing them from the soil or by immobilizing them or otherwise reducing their bioavailability. The latter approach is gaining increasing attention among researchers and practitioners as complete TE separation from soil is often technically challenging and rarely achievable.

Chemical stabilization or immobilization of TEs is typically implemented by adding amendments to the soil, which are materials capable of inducing geochemical reactions that lead to the formation of strong bonds between the amendments and TEs or those that lead to changes in TE oxidation state.^1^ As a result, the solubility, mobility, and bioavailability of TEs decrease along with their toxicity.^2,3^ However, the presence of high concentrations of organic pollutants, such as petroleum hydrocarbons, may interfere with these mechanisms. For example, organic compounds may occupy or block the surface of amendments, reducing their effectiveness in immobilizing TEs. Thus, the co-occurrence of multiple contaminants can introduce mutual limitations for several remediation strategies that might otherwise perform effectively in the presence of a single type of contaminant.

This issue is particularly prevalent at former wood treatment sites, where creosote and chromium–copper-arsenic (CCA) preservatives were often used sequentially over time to protect timber (mainly) from biological degradation. Soils and wastes at such sites commonly contain elevated levels of metals (e.g., copper (Cu), zinc (Zn), chromium (Cr)), metalloids (e.g., arsenic (As)), and polycyclic aromatic hydrocarbons (PAH), which may even be present in free-phase creosote.^4,5^ Alternatives to excavation and off-site treatment or landfilling of such soils/wastes include, e.g., *in situ* thermal treatment, chemical oxidation, and soil washing.^1,6^ However, these methods can be equally disruptive to soil health and are often limited in their capacity to address both organic and inorganic contaminants simultaneously.

Recently, a novel method for *in situ* trace element immobilization has been tested in laboratory settings and at pilot scale in the UK and Sweden.^7–9^ In these studies, corrosion products of iron (Fe) electrodes were used as a source of Fe amendments to soil, which were spread through the soil profile by a low-current electrical field. Building on this approach, the present study evaluated the method’s application to cocontaminated soil, investigating its ability to degrade PAH via electrochemical oxidation while simultaneously immobilizing As.

The aim of this study was to assess the efficiency of electricity-induced As immobilization and simultaneous PAH degradation *in situ* by monitoring changes in their dissolved concentrations in groundwater on a pilot scale at a former wood impregnation site in Sweden. Additionally, the study aimed to evaluate potential side effects of the treatment, including changes in As speciation and accumulation of PAH degradation products in groundwater, along with alterations of microbial community structure that might reflect changes in groundwater status or biogeochemical functional potentials.

## 2 Experimental Section

### 2.1 Pilot Site

The pilot study was conducted at a former wood treatment site in Limmared, Tranemo municipality, Sweden (57.5375° N, 13.3555° E). Pressure impregnation was primarily conducted to treat railway sleepers on-site using creosote oil and Boliden salt (containing Cu/Zn, Cr, and As). Treatment operations took place over an area of approximately 33,000 m^2^. As a result, the site has been extensively contaminated with high concentrations of As and creosote oil (PAH), affecting the soil, groundwater, and ditch sediments. The plot for the current pilot study was selected based on data from previous investigations.^10^ The location of a hot spot with high concentrations of As and PAH at relatively shallow depths (ca. 2 m below the surface) was identified in the northeast part of the site (where impregnation activities were carried out) as suitable for the pilot. The soil profile of the pilot area, in common with the rest of the site, contains a peat layer present at varying soil depths ranging from approximately 0.1 to 2 m below ground level. Beneath the peat, and in some places above it, lies a sand deposit, known as the Tranemo-Limmared Formation.^10^ At several locations in the pilot site the peat and sand layers were intermixed, likely due to previous construction activities, such as excavation for concrete footings, environmental investigations using pit excavations, and sampling (Table S1). Shallow groundwater at the site predominantly flows southward, although variations occur, and a hydraulic gradient toward the north has also been observed. Both the hydraulic gradient and groundwater flow velocity at the site are generally considered low.^10^ According to previous investigations, contaminant concentrations in soils at the pilot site were 35 800 mg kg^–1^ for PAH and 513 mg kg^–1^ for As (at 0.85–2 m depth).

### 2.2 Installations for Soil Treatment

The pilot test was implemented over an area of ca. 450 m^2^ (30 m × 15 m). Iron reinforcement rods with dimensions of Ø35 mm × 2.1 m were used as electrodes. In total, 28 electrodes were placed 5 m apart in a grid pattern (Figure S1) and driven into the ground using a geotechnical drilling rig to approximately 0.1 m above the surface, enabling the lower part of the electrode to reach a depth of ca. 2 m (covering the contaminated soil layer). PVC insulated Cu cables were used to connect the electrodes to each other and with a control unit. The control unit for the current distribution was provided by Ekogrid Oy (Finland) and placed at the edge of the experimental area. The setup was based on EKOGRID technology (Ekogrid Oy) originally designed to initiate electrochemical oxidation of organic pollutants and enhance bioremediation processes. A low-voltage DC current (10.5 V, 2 A) was distributed through the electrodes in pulsating mode with alternating polarity, enabling all electrodes to operate as anodes and cathodes. Such design allows for creation of multiple anode/cathode microareas within the treated site allowing for electrochemical reactions to occur throughout the bulk soil. The experiment was conducted for 2 years (775 days).

Six dense polyethylene groundwater wells were installed to a depth of 2.5 m. Four wells (GW1, GW2, GW3, and GW4) were placed within the experimental plot, one well (GW Ref1) was positioned upstream, and one well (GW5) was positioned downstream of the plot (Figure S1). Additionally, an older groundwater well from previous site investigations was identified within the plot, located adjacent to GW4, but no sampling in this well was implemented during the project. Groundwater PAH concentration data from this older well, collected during the four years prior to the experiment, were used for comparison.

### 2.3 Groundwater Sampling

Groundwater samples were collected once a month during the first year and every third month during the second year (July 2022 to August 2024) by using a peristaltic pump. The groundwater level was measured prior to each sampling event. One well volume was purged and allowed to fully recover before the sample collection. Samples for PAH analyses were collected into dark glass bottles and for metals into acid-washed polyethylene (PE) bottles. Sampling and analysis of groundwater at point GW5 were discontinued due to the presence of a creosote free-phase layer, which limited the representativeness of the samples. For those few samples that were collected, precautions were taken to prevent oil from entering the sampler, but creosote droplets were still observed in these samples.

Sampling for microbial and fungal analyses was conducted on three occasions, representing different seasons: autumn (September 2023, day 180), winter (January 2023, day 432), and early summer (June 2024, day 679). Groundwater was collected in 1 L PE bottles. Prior to sampling, the wells were flushed once to replace stagnant water, and the initial outflow was used to rinse the bottles. For the sample containing a free oil phase (GW5), care was taken to avoid collecting oil droplets. All samples were transported to the laboratory in a cooling box. The groundwater was then filtered through 0.2 μm nitrocellulose membrane filters under a fume hood, with the filtration device covered to minimize airborne contamination. Residual particles adhering to the inner walls of the device were rinsed with deionized water to ensure complete transfer to the filter. Filters containing the retained material were placed in sterile Petri dishes and stored at – 20 °C until shipment for analysis in a cooling bag.

### 2.4 Groundwater Analyses

Groundwater was analyzed for pH, redox, and electrical conductivity immediately upon arrival at the laboratory. All samples were filtered through 0.45 μm nitrocellulose membrane filters and acidified with concentrated nitric acid prior to analysis of metal concentrations using ICP-OES (PerkinElmer Optima 8300). Arsenic speciation in groundwater was performed by HPLC-ICP-MS according to SOP-0601 at ALS Scandinavia, Sweden, ISO/IEC 17025. The method is based on ion chromatographic separation of As species using an anion-exchange column, followed by ICP-MS detection. Detection limits for As­(III), As­(V), and DMA were 0.1 μg L^–1^, and for MMA they were 0.2 μg L^–1^. Analysis of PAH was carried out by ALS Scandinavia, Sweden, using headspace GC-MS in accordance with EPA Method 5021A Rev. 2, Update V. Measurement uncertainty for substances detected above the reporting limit was expressed as expanded uncertainty in accordance with JCGM 100:2008,^11^ using a coverage factor of 2 (≈95% confidence level). The provided reporting limits (LOR) were standard and specific for each parameter in the analytical method. Quality control included method blanks, laboratory control samples, laboratory duplicates, and surrogate standards, at a frequency of about one per 20 samples, with recoveries generally required to fall within 70–130% and duplicate precision assessed by relative percent difference (RPD) against method-specific limits.

Concentrations of oxygenated and nitrogen-containing PAH were analyzed by an accredited laboratory PiCA Prüfinstitut Chemische Analytik GmbH, Germany, DIN EN ISO/IEC 17025. The method is based on sample extraction and cleanup, followed by gas chromatography coupled with tandem mass spectrometry (GC-MS/MS). Detection limits for each compound are provided in Table S2.

### 2.5 Groundwater Microbiome Sequencing

Microbial communities, prokaryotic and fungal, were examined via 16S and internal transcribed spacer (ITS) rRNA gene sequencing, respectively. DNA was extracted from sample filters by using FastDNA SPIN Kit for Soil (standard or 50 mL formats; MP Biomedicals) according to the manufacturer’s instructions with the following amendments: (1) initial sample homogenization was achieved by a 15 min bead-beating step at maximum speed with a Vortex-Genie 2 vortex mixer (Scientific Industries, Inc.) and Vortex Adapter (QIAGEN), and (2) DNA samples extracted with the 50 mL kit format were each eluted into 1 mL of TES buffer and then concentrated into 50 μL of TES with a Zymo Genomic DNA Clean & Concentrator-10 Kit (Zymo Research) to ensure sufficient concentration for downstream sequencing. DNA extracts were quantified with a Qubit 1X dsDNA High Sensitivity (HS) Assay Kit (Thermo Fisher Scientific). They were then PCR-amplified for the prokaryotic 16S rRNA gene at the V3–V4 region with the Pro341F-Pro805R primers (5′-CCTACGGGNBGCASCAG-3′ and 5′-GACTACNVGGGTATCTAATCC-3′, respectively) and for the fungal ITS2 subregion with primers gITS7 ngs-ITS4 ngsUni (5′-GTGARTCATCRARTYTTTG-3′ and 5′-CCTSCSCTTANTDATATGC-3′, respectively). After an initial 25-cycle PCR, Illumina adaptors were ligated via a 10-cycle PCR. Amplicons were purified with an Agencourt AMPure XP PCR cleanup kit (Beckman Coulter), quality-assessed via a D1000 DNA ScreenTape assay on a 4200 Tapestation (Agilent), and had their concentrations measured with a Qubit dsDNA High-Sensitivity assay kit (Thermo Fisher Scientific). Purified amplicons were pooled at equimolar concentrations of 4 nM for library preparation with a Nextera XT DNA kit (Illumina) and sequenced on an Illumina MiSeq platform (M02946, Illumina). Three negative control samples were subjected to all extraction and sequencing steps.

Sequence demultiplexing and adapter trimming were conducted directly after sequencing with MiSeq control software (Illumina). Sequence quality assessment and filtering, primer removal, amplicon sequence variant (ASV) inferral, merging, chimera removal, and taxonomy assignment were performed via the standard DADA2 pipeline (v1.14 ^12^), with databases SILVA (v138.2 ^13^) and UNITE (v10.0 ^14^) for prokaryotic 16S rRNA and fungal ITS, respectively. For downstream analysis, ASVs not assigned as bacteria or archaea in 16S libraries were removed from analyses. ASVs not classified as fungi were removed from ITS2 libraries.

### 2.6 Statistical and Microbial Diversity Analysis

For physicochemical data, *Statgraphics Centurion 19* software was used for analysis of variance (ANOVA), multifactor ANOVA, and correlation analysis. A two-sample *t*-test (*p* < 0.05) was used to discriminate between sample means.

For sequencing data, all diversity and statistical analyses were performed by using R statistical software (v4.4.0 ^15^) with ggplot2 (v3.3.5 ^16^) for graphical representation and packages Microeco (v1.15.0 ^17^), vegan (v2.5.7 ^18^), and phyloseq (v1.26.0 ^19^) for community diversity analyses. All statistical tests performed were subjected to false discovery rate *p*-value adjustment, with adjusted *p-*values of <0.05 considered significant.

Alpha-diversity (intracommunity diversity) was examined with Chao1 (community richness) and Shannon’s (community richness and evenness) indices calculated for each sample. Their differences between sample locations and dates were assessed by Kruskal–Wallis tests, while Spearman’s rank correlation was used to test for associations with physiochemical factors. Beta-diversity analysis based on Bray–Curtis dissimilarity was performed to compare community structure between samples, after normalization via scaled ranked subsampling (SRS)^20^ to account for variability in library sizes between samples. Marginal permutational multivariate analysis of variance (PERMANOVA; 9999 permutations; vegan) was used to assess the variance explained by sample location, date, and physicochemical factors.

Correlations between the relative abundance of individual taxa with physiochemical factors were assessed via Spearman’s rank test, while linear discriminant analysis effect size (LEfSe) was calculated to identify taxa significantly enriched in samples of high As and PAH concentrations (>1 mg L^–1^) – with a linear discriminant analysis (LDA) value of >3.5 rendering taxa enrichment.

## 3 Results and Discussion

### 3.1 Groundwater Levels

Groundwater level fluctuation corresponded to variations in wet–dry seasons (Figure 1). The highest levels were observed during late autumn through spring (max reaching 34 cm below the surface), followed by a decrease during summers (max reaching 205 cm below the surface). This indicates that the inserted electrodes were always submerged into the groundwater, which facilitated distribution of electrical current and ensured that the method had favorable prerequisites to function. Sufficient soil humidity or water content is essential for this method to work.

**Figure 1:**
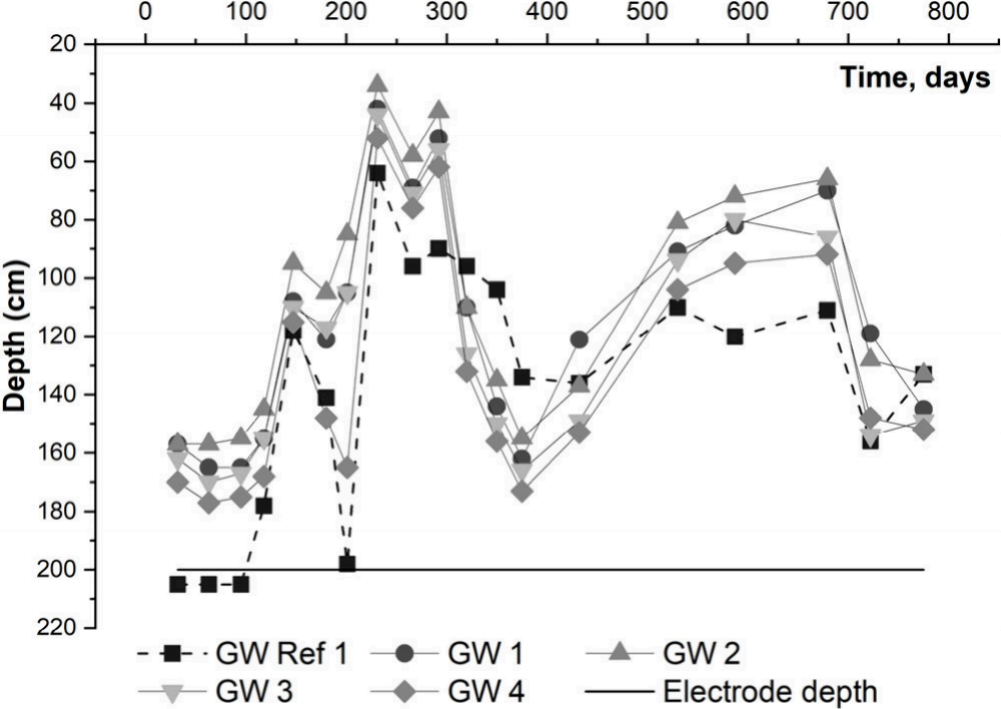
Fluctuation of the groundwater level at the sampling points of the pilot field test site.

### 3.2 Electrical Conductivity, pH, and Redox Potential in Groundwater

Electrical conductivity (EC) fluctuated over time but remained relatively stable when comparing initial and final values (Figure 2A). The lowest conductivity was observed at point GW4 (168 μS cm^–1^), whereas all the other sampling points ranged between 201 and 250 μS cm^–1^. Typically, during electrochemical treatment, especially when using a one-directional current (DC), EC tends to decrease due to the migration and possible depletion of ions in the treated area. However, the rather stable EC in this experiment suggests that there was a continuous presence of dissolved ions in groundwater, likely maintained by adequate moisture levels and possibly ongoing ion exchange or mineral dissolution processes. This stability may indicate that the electrochemical process did not significantly deplete available charge carriers in the system, allowing for sustained conductivity over time.

**Figure 2:**
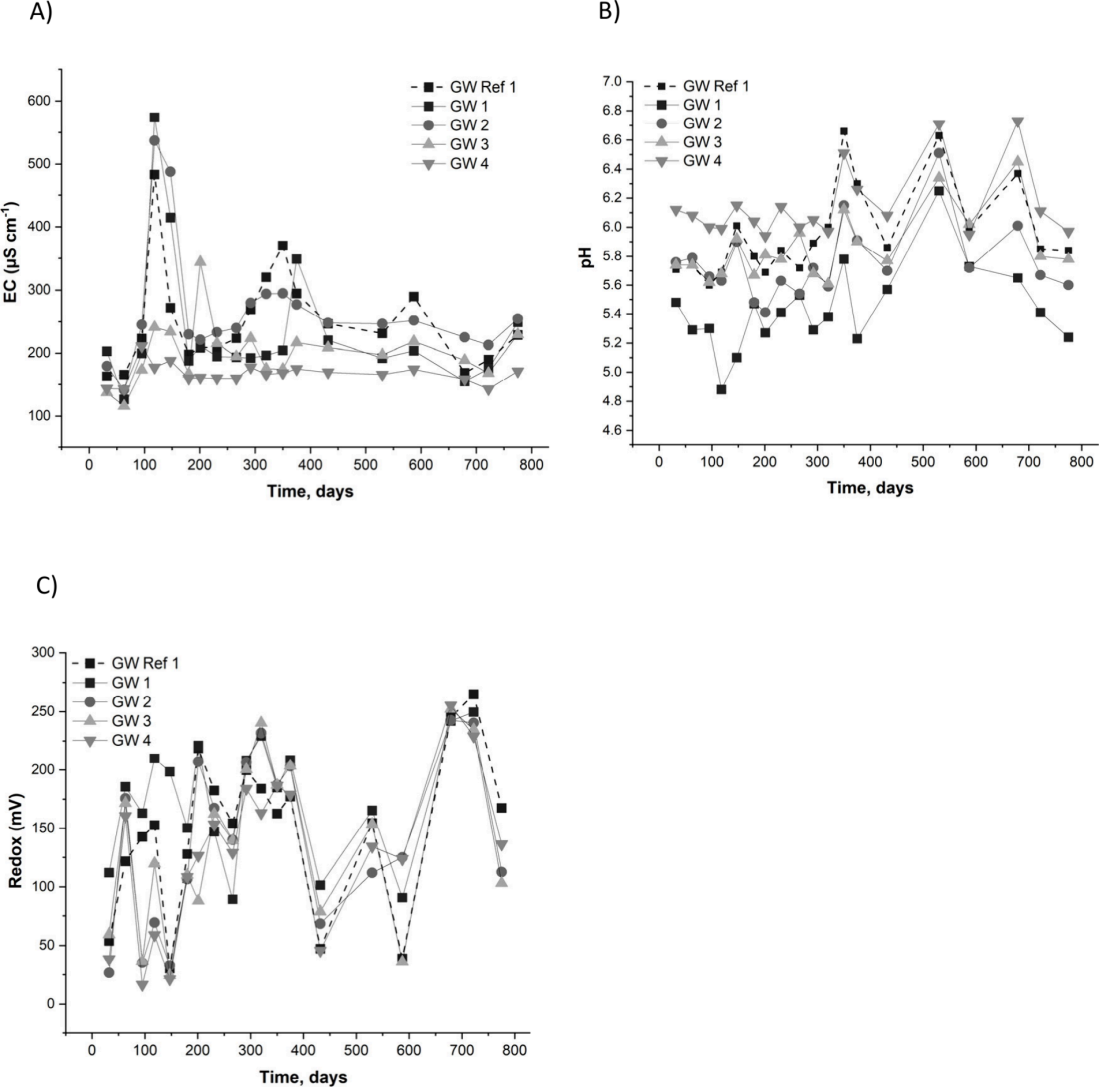
Variation in a) electrical conductivity (EC), b) pH, and c) redox potential in groundwater during the field experiment.

Groundwater pH values fluctuated slightly throughout the experiment but remained within a relatively narrow range (Figure 2B). The average pH across all sampling points over the entire experimental period was 5.85 ± 0.34. This stability was expected given the application of electrical current with reversing polarity. Traditional electrokinetic treatments using one-directional current often lead to significant pH gradients - acidification near the anode and alkalization near the cathode - due to electrolysis reactions.^21^ Such pH shifts can negatively affect soil properties, including microbial activity, which is particularly important when biodegradation of organic pollutants is desired. In contrast, the use of pulsed current with reversed polarity in this experiment appears to have mitigated extreme pH changes. This suggests that the applied electrochemical conditions maintained a more balanced soil environment, potentially preserving its biological and chemical stability.

Redox conditions play a crucial role in As immobilization by using Fe compounds. A significant decrease in the redox potential can trigger the reductive dissolution of Fe,^22^ potentially releasing soil-bound As back into the environment. In our previous laboratory studies on the electrochemical immobilization of As in the same organic soil, a notable drop in redox potential was observed. However, in this field experiment, despite some fluctuations, the redox values remained above zero (Figure 2C). This stability suggests that oxygenated conditions were maintained throughout the experimental period despite the presence of high amounts of natural organic matter, which typically promotes reducing conditions. The sustained positive redox potential is favorable for As immobilization and is advantageous for microbial communities, particularly aerobic bacteria that play a key role in the degradation of PAH.

There was no significant correlation between any of the above-discussed variables, i.e., groundwater depth, pH, redox potential, or EC.

### 3.3 Arsenic in Groundwater

Averaging the results over the course of the experiment, a general decrease in dissolved As concentrations was observed across most sampling points compared to initial values (Figure 3). However, concentrations fluctuated over time, with periodic peaks occurring at all points (Figure 4). GW1 exhibited the lowest As concentrations, remaining within the range observed at the reference point, and a decrease over time was insignificant. Mean values over the two years for GW2–4 differed significantly from the initial values. GW2 showed the clearest decrease in dissolved As (by 88%), despite occasional peaks (Figures 4 and S3). The treatment appeared to have less significant impact on dissolved As concentrations at GW3 and GW4 (which decreased by 55% and 57%, respectively), and values fluctuated substantially, particularly in GW3, resulting in a large standard deviation of the means (Figure 3). Notably, both GW3 and GW4, especially GW3, also had high concentrations of PAHs (Figure 5). This suggests that the immobilization of As may have been hindered at these points compared with GW2, where PAH concentrations were close to reference levels. There was a positive correlation between As and PAH concentrations in groundwater in GW2, meaning that decreasing PAH concentrations also showed a decrease in dissolved As concentrations. However, such a correlation was not significant in GW3 and GW4, indicating that PAH and As concentrations varied independently of each other, most likely because PAH concentrations were much higher in these points, particularly in GW3, compared to GW2 (Figure 4). The peat-rich zones in GW3 and GW4 likely introduced a higher heterogeneity into the sorption properties. In such environments, Fe oxyhydroxides may be partially coated or complexed by organic compounds, reducing their effective surface area and thereby limiting Fe–As interactions compared with the more mineral-dominated zone at GW2. Also, high organic matter content can promote formation of soluble organometallic complexes that increase As mobility.^23^ This suggests that effective As immobilization may rely on PAH degradation occurring first, as reducing the organic load creates more favorable conditions for As to sorb onto Fe phases.

**Figure 3:**
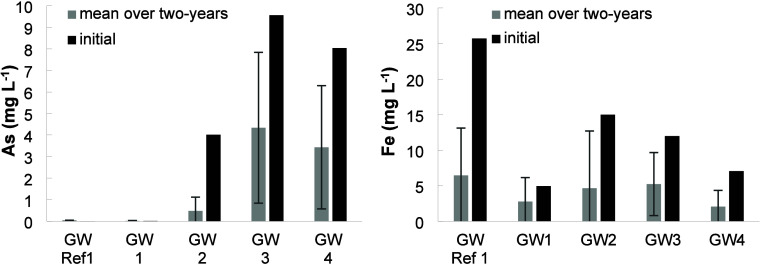
Mean As and Fe concentrations in groundwater over the course of the experiment in comparison to the initial values measured at the start of the experiment. Note: As concentrations in GW Ref1 and GW 1 were below instrument detection limits.

**Figure 4:**
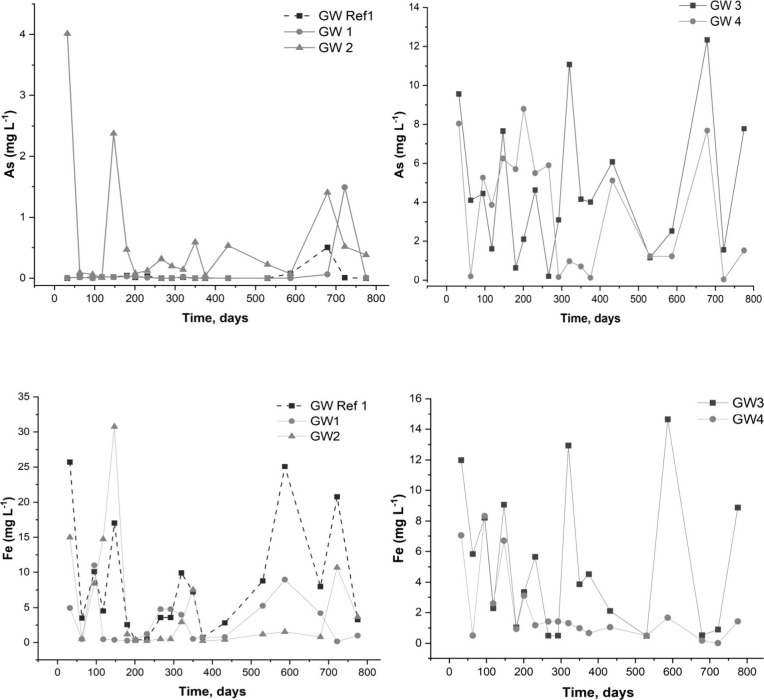
Variation in As and Fe concentrations in groundwater wells over the two years of the experiment.

**Figure 5:**
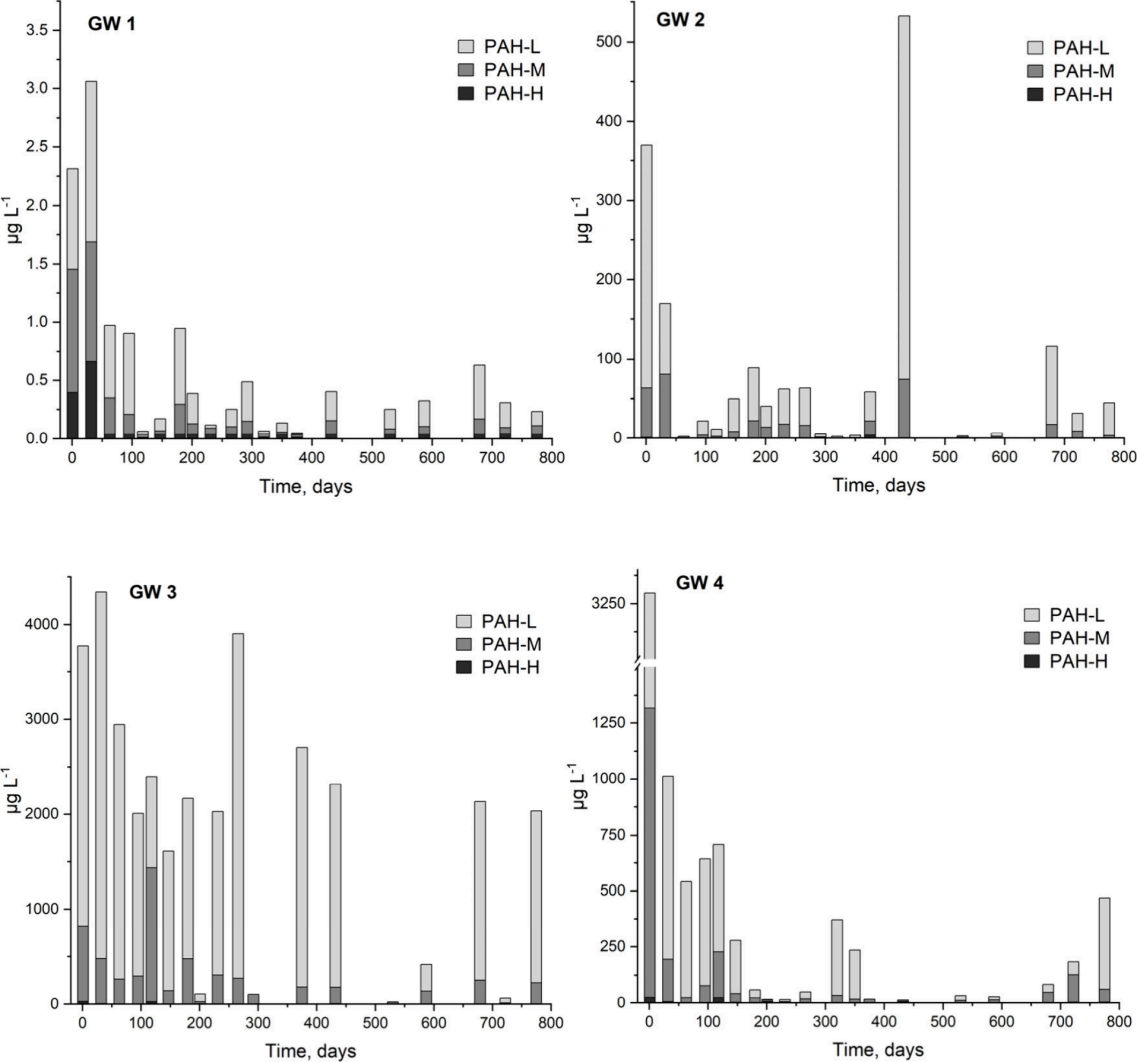
Variation of PAH concentrations divided into low (PAH-L), medium (PAH-M), and high (PAH-H) molecular wight fractions in four groundwater wells at the experimental plot over the duration of two years. Note the change in *y*-axis scales. Concentrations in groundwater of the reference point (GW Ref1) were largely below instrument detection limits and therefore excluded from this figure.

The speciation of dissolved As in groundwater measured on three occasions during the second year of the experiment showed the presence of minor amounts of the reduced arsenite species, with no detectable monomethylarsonate (MMA) and, on rare occasions, very low concentrations of dimethylarsinate (DMA), ranging from 0.17 to 2.85 μg L^–1^ (Figure S2). Dissolved As was predominantly present in its less toxic form, pentavalent arsenate, accounting for 96–100% of total As, except for one instance where this species constituted 82%. The dominance of arsenate is generally favorable, as it suggests that potential As toxicity has not increased by methylation, even in the presence of high soil organic matter content at varying depths (Table S1) and potential stimulation of bacterial activity by the low-voltage electrical field.^24^ Moreover, this observation aligns with the consistently positive redox values recorded throughout the experiment.

### 3.4 Iron in Groundwater

The concentration of dissolved Fe in groundwater, similar to As, fluctuated substantially over the treatment period (Figures 3 and 4). Notably, dissolved Fe concentrations were highest in the reference point (GW Ref1), with a two-year average of 7.6 mg L^–1^, compared to the other sampling points, where the average values ranged between 2.4 and 5.6 mg L^–1^. The fluctuating patterns of dissolved Fe concentration closely mirrored those of As (Figure 4), and correlation analysis revealed a statistically significant relationship between dissolved Fe and As in all points except the reference (GW Ref1). The strongest positive correlation was observed in GW3 (coefficient of 0.69), followed by GW2 (0.66) and GW4 (0.58). In contrast, GW1 showed a moderate negative correlation (−0.50), indicating that as Fe concentrations increased, As concentrations decreased and vice versa. Notably, the strongest positive correlation was found at GW3, where the highest PAH concentrations were observed. This further strengthens the earlier reasoning that organic compounds may have influenced Fe–As interactions, possibly hindering the reactions between Fe and As while keeping Fe in groundwater likely bound to dissolved OM. This underscores the challenge of identifying soil remediation techniques capable of addressing both types of contaminants simultaneously. It is likely that Fe–As coprecipitation or other interactions between Fe and As will become more pronounced as PAH concentrations decline over time. Therefore, ensuring the rapid degradation of PAH might be essential to facilitate the faster immobilization of As.

### 3.5 PAH in Groundwater

The distribution of PAH based on their molecular weight showed that low-molecular-weight PAH (PAH-L) accounted for 55–84% of dissolved PAH present, followed by medium-molecular-weight PAH (PAH-M) with an average presence of 15–31%, and high-molecular-weight PAH (PAH-H) contributing 0.4–13% of the total PAH. Although PAH-M compounds typically dominate in creosote oil and creosote-contaminated soils,^4,25^ lower molecular weight PAH have higher water solubility and so are expected to show higher concentrations in groundwater than heavier PAH.

Dissolved concentrations of PAH in groundwater decreased over time across all monitoring wells. Some intermittent spikes were observed, with the most significant occurring at a single instance in GW2 and GW3 (Figure 5). Over the two-year treatment period the average reduction in dissolved PAH concentrations, relative to initial levels, was substantial at all sampling points, reaching 85% in GW1, 84% in GW2, 62% in GW3, and 94% in GW4. The greatest average reduction was observed for PAH-M at 84%, followed by PAH-H at 76%, and PAH-L at 74%. Since PAH-H concentrations were low initially, the observed decrease resulted in even lower or negligible concentrations of these compounds (0.06–2.7 μg L^–1^) by the end of the experiment. These findings align with our previous observations that electricity-stimulated oxidation of PAH occurs simultaneously across all molecular weight fractions,^25^ in contrast to bioremediation, which predominantly facilitates the degradation of lighter PAH.^26^

Degradation of high-molecular-weight PAH generally does not result in the formation of intact lower-molecular-weight PAH. Instead, oxidative (bio)­transformation predominates, leading to the formation of oxygenated intermediates (oxy-PAHs, ring-cleaved acids, etc.).^27,28^ For example, during biotransformation, microbial and enzymatic pathways usually initiate an oxidative attack on aromatic rings, forming oxygenated intermediates such as dihydrodiols, phenols, and quinones that are further processed through catabolic sequences.^29^ Therefore, the observed intermittent spikes in PAH concentrations, primarily consisting of low-molecular-weight PAHs (PAH-L), are most likely attributable to desorption from soil and subsequent resupply into the groundwater. This process may also explain why the total PAH-L degradation was not the highest among the molecular weight fractions. While substantial PAH-L degradation occurred over time, continuous resupply from the soil to the aqueous phase maintained high concentrations of these compounds in groundwater. In addition, transient increases may have been influenced by electrochemically induced mass transport (e.g., electroosmosis or electromigration) as well as, to a lesser extent, fluctuations in groundwater flow or redistribution of residual NAPL phases.

Analysis of the available data collected prior to the start of the experiment from the older groundwater well adjacent to GW4 indicates that PAH concentrations varied substantially - by more than an order of magnitude - between 2018 and 2021 (Figure S4).^30^ The mean PAH concentration during that period was 3674 μg L^–1^. The initial concentration of PAH in GW4 was 3296 μg L^–1^, which is very close to this four-year average. Shortly thereafter, the PAH concentration in GW4 dropped to 1012 μg L^–1^ and continued to decline, although with some smaller fluctuations (Figure 5). These results suggest that the soil treatment for this point was effective, and given more time, the concentrations would likely have decreased further. It is also noteworthy that the characteristic smell of volatile creosote compounds, which was strongly noticeable at the beginning of the experiment, gradually declined and was often not detectable at all in this sampling point.

### 3.6 Oxygenated and Nitrogen-Containing Heterocyclic PAH

Concentrations of ten oxygenated (oxy-PAH) and three nitrogen-containing heterocyclic PAH (N-PAH) were measured at five occasions during the second year of the experiment (Table S2). Out of 13 measured compounds, four (acridine, 1-indanone, carbazole, and 9-fluorenone) were measured at tens of μg L^–1^, one, quinoline, was measured at a few μg L^–1^, and the remaining were present at concentrations below microgram levels (Table S2). Thus, the concentration ratios of detected transformation products to parent PAH were very low. Although oxy-PAH and N-PAH compounds are typically abundant in soil at creosote-contaminated sites,^31^ they were not detected in significant amounts in the groundwater at the studied site. This suggests that transformation products, which are more water-soluble than their parent PAH, underwent continuous degradation and did not accumulate. These findings are reassuring, as they indicate that electrochemical oxidation of PAH was unlikely to cause accumulation of secondary pollutants. In contrast, Eriksson et al.^32^ observed an accumulation of 4-hydroxy-9-fluorenone in biotreated PAH-contaminated soil, exceeding the concentration of its parent PAH (fluorene) by 550%. Although this appears to be an extreme case, other studies have also reported accumulation of oxy-PAH concentrations in biotreated soils ranging from 10% to 68% of the original PAH concentrations.^33–35^

### 3.7 Microbial and Fungal Communities in Groundwater

Prokaryotic communities showed the highest alpha diversity at GW1, as indicated by both species richness (Chao1) and evenness (Shannon), followed by GW Ref1, GW2, then GW3, and GW4 on average (Figure 6A). This general decline in alpha-diversity with increasing As and PAH concentrations, despite lack of significant correlation, is consistent with the typical negative impacts of metal and organic contamination on microbial diversity.^36^ At GW1, both diversity indices in fact increased over time (Figure 6A). There was no significant relationship, however, between alpha diversity and other measured physicochemical parameters (pH, redox potential, conductivity), suggesting that additional factors influence prokaryotic diversity across sampling sites.

**Figure 6:**
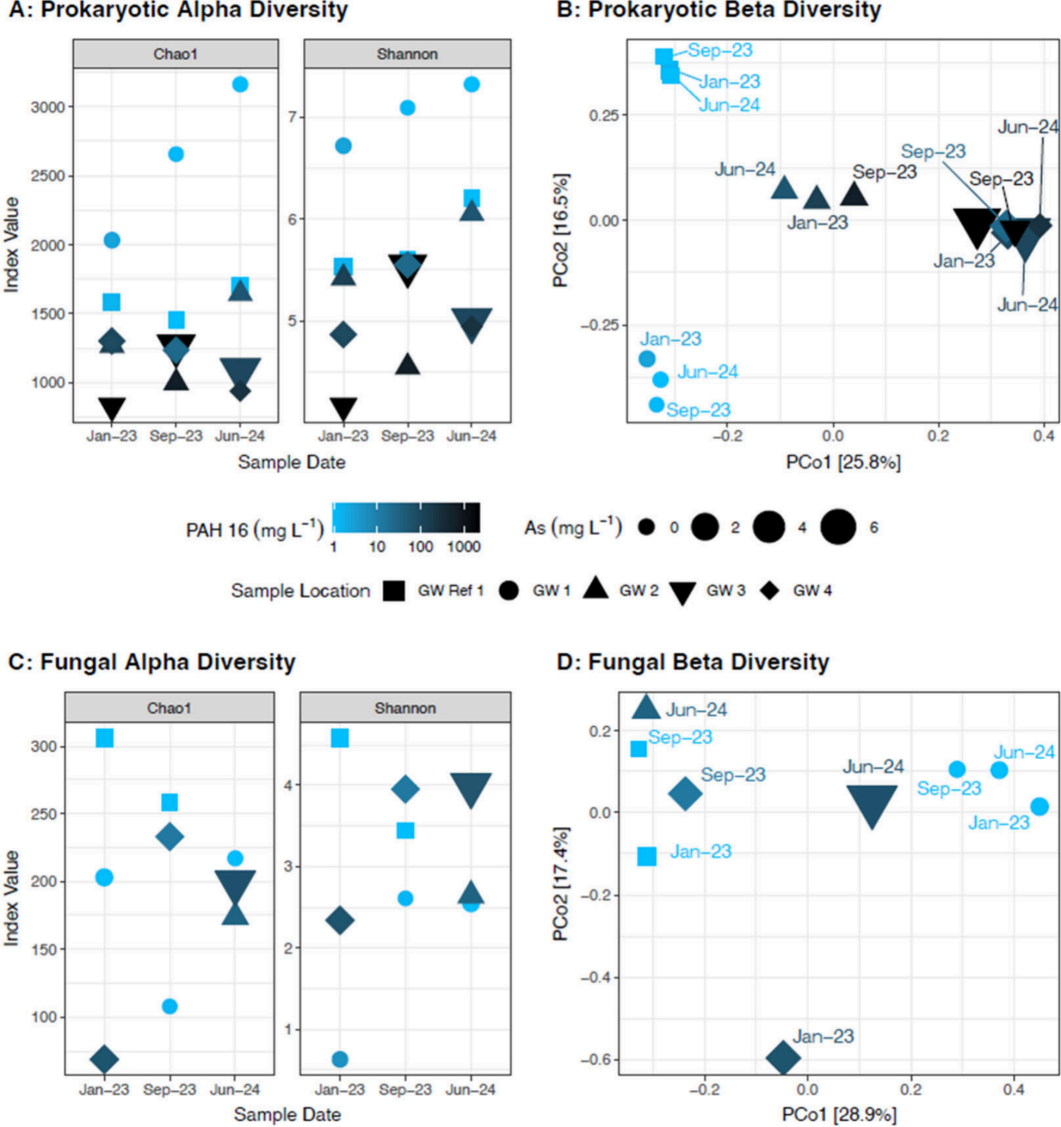
**Alpha and beta diversity of microbial communities across groundwater samples.** Plots A and B show alpha and beta diversity of prokaryotic (16S) communities, respectively. Plots C and D show alpha and beta diversity of fungal (ITS2) communities, respectively. Alpha diversity is represented by Chao1 (community richness) and Shannon’s (community richness and evenness) indices. Beta diversity is shown via principal coordinate analysis (PCoA) based on Bray-Curtis dissimilarity of samples. The legend is representative of all plots, with As concentrations for each sample indicated by the point size, PAH concentrations indicated by the point color, and sample location indicated by the point shape.

In comparison, fungal alpha diversity displayed less consistent patterns (Figure 6C), though fungi were only detectable in 9 out of 15 samples. The highest fungal diversity occurred at GW Ref1, peaking in January 2023 (day 180); however, this was also when the lowest values were recorded at GW4 for Chao1 and at GW1 for Shannon. There were no observable trends between contaminant concentrations and fungal alpha-diversity. The relatively small number of successfully sequenced fungal libraries likely constrained the detection of robust trends.

Beta-diversity analysis based on Bray–Curtis dissimilarity and principal coordinate analysis (PCoA) showed clear spatial differences of prokaryotic community structures between the wells (Figure 6B), as supported by marginal PERMANOVA (R^2^ = 0.32, p = 0.003). Prokaryotic communities at GW1 and GW Ref1, where contaminant concentrations were lowest, were particularly distinct from the rest, while communities at GW3 and GW4, where As and PAH concentrations were highest, clustered together. The sampling date did not significantly influence prokaryotic community structure. While GW1 fungal assemblages formed a visually distinct cluster similar to prokaryotes, fungal communities overall showed weaker clustering patterns (Figure 6D), and PERMANOVA indicated no significant site difference.

Across all sampled wells, Gammaproteobacteria dominated the prokaryotic communities (11–59%; Figure 7A), followed by Alphaproteobacteria or Bacteroidia. An exception occurred at GW1 during September 2023 (day 432) and June 2024 (day 679), when Parcubacteria predominated (18–20%). Parcubacteria belong to the Patescibacteria superphylum and are noted for their small, streamlined genomes and adaptation to groundwater environments.^37^ Other abundant classes included Actinobacteria, Verrucomicrobilia, and Campylobacteria, which are common in soil and groundwater.

**Figure 7:**
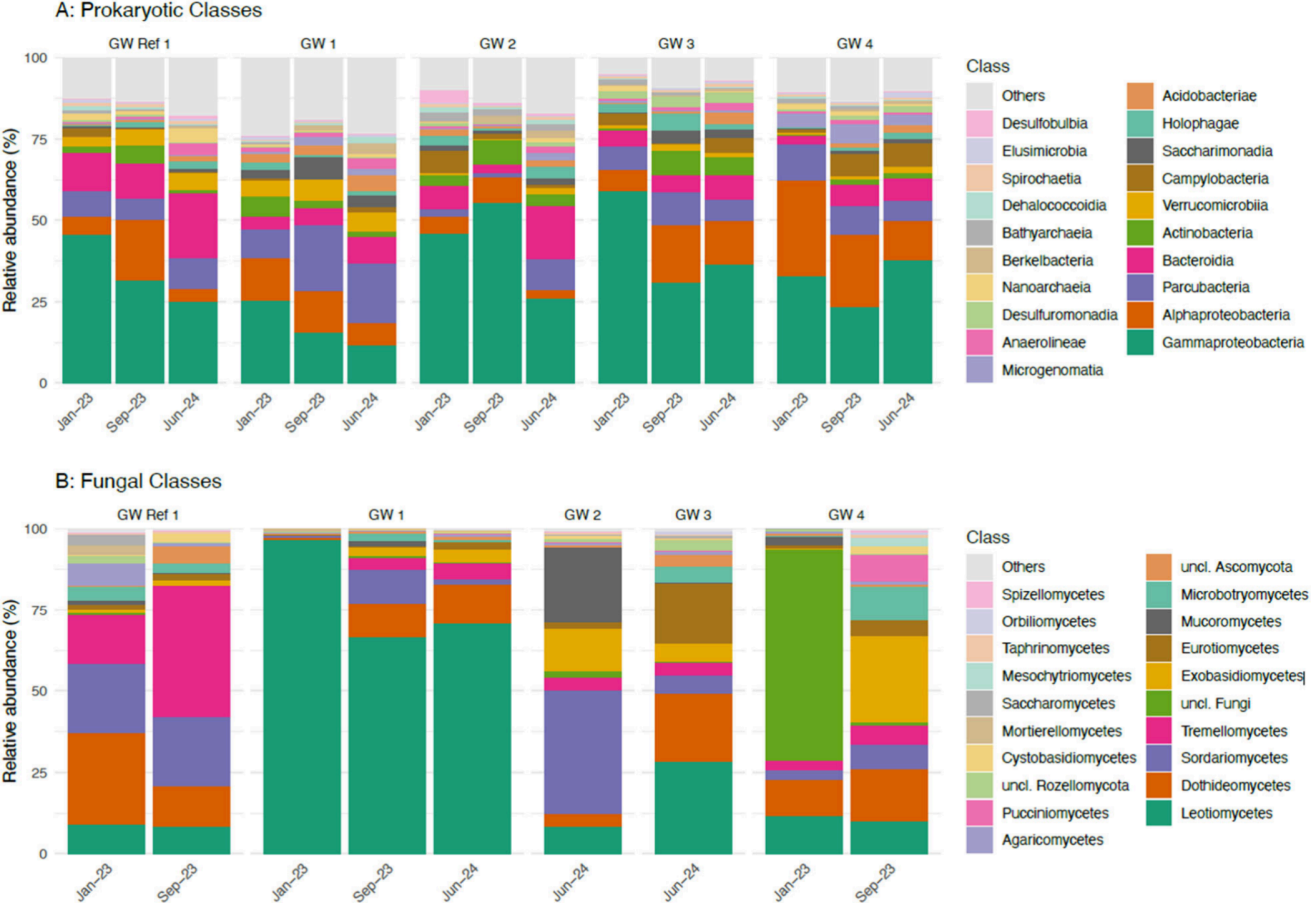
**Prokaryotic and Fungal class relative abundance across groundwater samples.** Only the top 20 most relatively abundant prokaryotic (16S) and Fungal (ITS2) classes are shown, with the remaining classes pooled into the “Others” category. Note that fewer samples yielded amplified libraries for fungal communities.

At the family level, four of the ten most abundant taxa belonged to the order Burkholderiales: *Rhodocyclaceae*, *Comamonadaceae, Gallionellaceae*, and *Oxalobacteraceae* (Figure S7). Together with *Pseudomonadaceae* (Pseudomonadales) and *Sulfurimonadaceae* (Campylobacterales), these families generally increase in relative abundance with increasing As and PAH concentrations toward GW3 and GW4 – except for the Fe-oxidizing *Gallionellaceae* that was more abundant at GW Ref1 along with the methane- (or methyl-compound) oxidizing *Methylomonadaceae*, where there were high concentrations of Fe and mostly likely of methane also in the peat soil therein.

At the genus level, the top three prokaryotic genera, *Rugosibacter* (Rhodocyclaceae), *Pseudomonas* (Pseudomonadaceae), and *Rhodoferax* (Comamonadaceae), were consistently enriched at the more contaminated GW2-GW4 wells. *Rugosibacter* and *Pseudomonas* showed significant positive correlations with both As and PAH concentrations (Figure 8), and *Rugosibacter* was additionally enriched under high-PAH conditions (LDA = 4.7, p = 0.019). Species within *Rugosibacter* have previously been isolated from contaminated soils and shown to degrade aromatic compounds such as phenanthrene, pyrene, and petroleum hydrocarbons^38,39^ and can express extradiol dioxygenases to degrade mono- and polycyclic aromatic compounds.^40^

**Figure 8:**
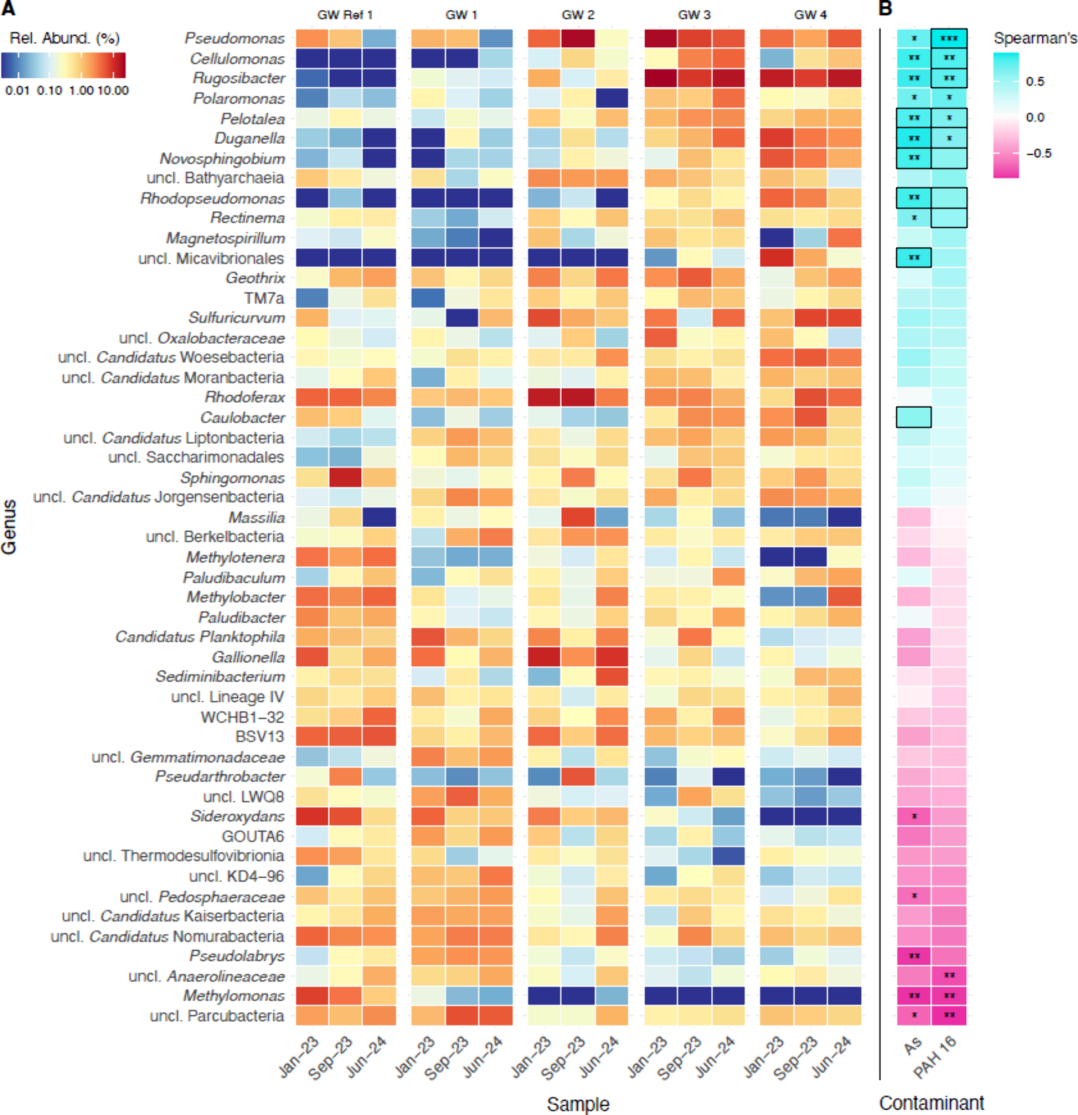
**Prokaryotic genera community compositions and associations with As and PAH contamination across groundwater samples.** A: Heatmap showing the relative abundance (%) of the top 50 most relatively abundant genera across samples. B: Heatmap showing key genera relative abundance Spearman’s rank correlations with As and PAH concentrations. Bold outlines indicate significant enrichment of corresponding genera in samples containing increased concentrations (>1 mg L^–1^) of indicated contaminants, as assessed by LEfSe analysis (*p* < 0.05, LDA > 3.5). Genera are sorted by their correlation with PAH, with those showing the strongest positive associations placed at the top of the plot. Only the top 50 most relatively abundant genera across samples are shown. Taxa preceded with uncl. could not be classified to the genus level and hence are assigned to the lowest possible taxonomic rank.

Several additional genera, including *Duganella*, *Pelotalea, Novosphingobium, Cellulomonas, Rhodopseudomonas, Polaromonas, Rectinema*, and unclassified members of *Cand*. Woesebacteria, *Cand*. Liptobacteria, and *Cand*. Micabivrionales, also showed significant positive correlations with As, and in some cases PAH concentrations (Figure 8). *Duganella* was particularly enriched, reaching 4.56 ± 2.93% at GW4 compared to 0.02 ± 0.02% at GW Ref1, and was significantly associated with both high As (LDA = 4.30, p = 0.021) and high PAH (LDA = 4.08, p = 0.019) concentrations in groundwater. Several of these genera, including *Pseudomonas*, *Rhodoferax*, *Duganella*, *Cellulomonas*, *Rhodopseudomonas*, and *Polaromonas*, are known to respire As.^41–43^ *Pseudomonas* and *Polaromonas* can additionally oxidize As­(III), and multiple *Pseudomonas* species are established PAH degraders.^44^ *Pelotalea* possesses Fe­(III)-reducing capabilities and has been isolated from petroleum-contaminated soils,^45^ suggesting potential indirect roles in As cycling, while *Rectinema*, an obligate fermenter within the Spirochaetes, is commonly found in hydrocarbon-contaminated groundwater.^46^

Although no significant correlations were detected, *Sulfuricurvum* and *Acidovorax*, both known to exhibit As tolerance,^47,48^ were consistently more abundant at the more contaminated wells (GW2, GW3, and GW4). *Acidovorax* is capable of oxidizing the more toxic arsenite [As­(III)] to arsenate [As­(V)] ^48,49^ and is also recognized for its ability to degrade complex aromatic organic compounds, including polychlorinated biphenyls (PCBs), pesticides, nitrobenzene, and phenanthrene.^50,51^

Notably, aerobic methane-oxidizing *Methylomonas* (and to a lesser extent *Methylobacter*), along with *Sideroxydans*, *Pseudolabrys*, unclassified *Parcubacteria*, and *Pedosphaeraceae*, were negatively correlated with As concentrations, while *Methylomonas* and *Parcubacteria* also showed negative correlations with PAH. This pattern may reflect inhibitory effects of contaminants or greater availability of labile organic carbon at GW1 and GW Ref1. Indeed, at GW Ref1, dissolved organic carbon concentrations were mostly higher than at other wells, while As and PAH levels were negligible. Iron­(II) oxidation by *Sideroxydans* and *Gallionella* has been shown to be stimulated by organic enrichment, including humic substances, leading to the formation of Fe­(III) oxyhydroxide nanoparticles.^52^ Such processes may contribute to As immobilization via sorption to Fe­(III) phases as suggested earlier.

Fungal community composition showed less coherent spatial structuring, though it is likely constrained by the fact that they were detectable only in 9 out of 15 samples. At the class level, Leotiomycetes (8–96%; Figure 7B), a group that includes many plant pathogens, dominated overall, particularly in GW1. Dothideomycetes (0.6–28%), Sordariomycetes (3–21%), and Tremellomycetes (0–40%) were widespread, with peak relative abundance at GW Ref1. In contrast, Exobasidiomycetes (0–26%) were most abundant at GW4 during September 2023, coinciding with high As and lower PAH concentrations, while Eurotiomycetes (0–18%) peaked at GW3, where both PAH and As concentrations were high. Mucoromycetes (0–23%) constituted a particularly large fraction at the single sample of GW2, where both As and PAH levels were moderate. No fungal families or genera showed any significant enrichment or correlations with respect to As or PAH contamination levels. The most abundant fungal genus, the lignin-degrading *Holwaya* (0–89%; class Leotiomycetes), was however present exclusively within the experimental plot (Figure 9).

**Figure 9:**
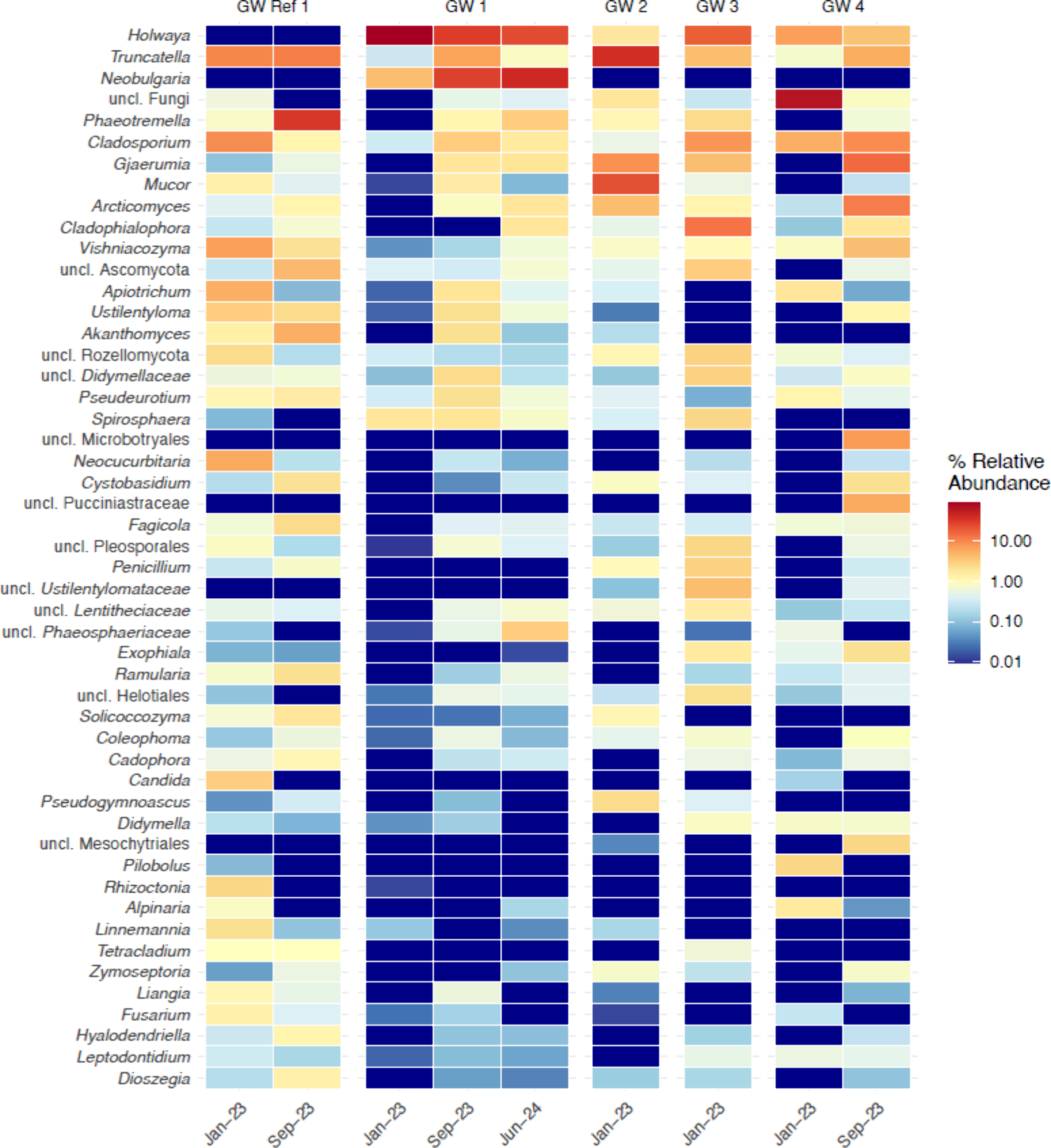
**Fungal genera community compositions and associations with As and PAH contamination across groundwater samples.** Heatmap showing the relative abundance (%) of the key fungal genera across samples. Genera are sorted by their correlation with PAH, with those showing the strongest positive associations placed at the top of the plot; note that none of the taxa shown exhibited significant correlations with As or PAH nor were significantly enriched in high (>1 mg L^–1^) contaminant samples. Only the top 50 most relatively abundant genera across samples are shown. Taxa preceded with uncl. could not be classified to the genus level and hence are assigned to the lowest possible taxonomic rank.

Among fungal genera with known bioremediation potentials (Figure S8), *Cladosporium* (1–9%), with members capable of degrading a range of aromatic compounds,^53,54^ was more abundant at GW3 and GW4, although it was also prominent at GW Ref1 in January 2023. Within Aspergillaceae, *Penicillium* (0–2%) showed modest increases in relative abundance at more contaminated sites, while *Aspergillus* was detected only at GW3 and GW4 though at trace levels (<1%) (Figure S8). Both genera include members with PAH- and As-transforming capabilities.^55^ Other fungi with reported relevance to PAH and As remediation, including *Pleurotus*, *Mucor*, and *Fusarium*,^53,55^ were generally more abundant in less contaminated wells, nonetheless.

### 3.8 Interactions between Electrochemical Processes, Fe Cycling, and Microbial Community Responses

The application of a pulsed low-voltage electrical current likely enhanced Fe cycling within the subsurface system by promoting the release of Fe^2+^ and Fe^3+^ from Fe electrodes while also potentially stimulating the dissolution of indigenous soil Fe phases.^56^ Although these two sources of Fe could not be distinguished experimentally, their combined contribution is environmentally relevant, as both pathways increase the availability of reactive Fe species. Once released, Fe^2+^ and Fe^3+^ undergo rapid hydrolysis and oxidation, forming Fe-oxyhydroxide phases such as ferrihydrite and goethite, which are well-known for their strong affinity for arsenate through inner-sphere complexation mechanisms.^23,57^

The formation of Fe-oxyhydroxides provides multiple stabilization pathways for As, including surface adsorption and coprecipitation during Fe oxidation. These processes substantially reduce As mobility, and structural incorporation into Fe mineral phases can further enhance long-term sequestration.^58–60^ In particular, arsenate forms stable bidentate binuclear complexes on ferrihydrite surfaces, which dominate under mildly acidic to circumneutral pH conditions and strongly control As partitioning between solid and aqueous phases.^61,62^

Groundwater geochemical conditions at the site supported the stabilizing processes. That is, consistently positive redox potentials (Eh > 0 mV) and stable pH values around 6 favor both Fe­(II) oxidation and the dominance of arsenate over the more toxic and mobile arsenite. Accordingly, arsenate accounted for more than 95% of dissolved As in most samples, indicating limited reductive remobilization. Redox conditions are a primary control on As mobility, as they regulate both Fe mineral stability and sorption capacity.^61^ Maintaining oxic conditions is therefore critical, since Fe­(III) reduction can dissolve Fe minerals and release previously immobilized As. From ecotoxicological and health risk perspectives, this is particularly relevant, given the substantially lower toxicity and mobility of arsenate compared with arsenite.^63^

Importantly, the microbial community structure observed in the site groundwater provides additional insights into process interactions. Along increasing As and PAH gradients, microbial communities showed clear selection for taxa tolerant of, and in some cases capable of transforming, elevated contaminant concentrations. This pattern was supported both by significant correlations with As and PAH concentrations and by differential enrichment identified through LEfSE analysis, indicating that contaminant pressure acted as a strong ecological filter on community composition.

In contrast, taxa associated with Fe­(II) oxidation, including *Gallionella* and *Sideroxydans*, as well as methanotrophic and methylotrophic groups, displayed opposing abundance trends, being more prevalent at less-contaminated sites. This divergence highlights a potential trade-off between contaminant tolerance and Fe-oxidizing functionality. Because Fe-oxidizing microorganisms contribute to the formation of nanoparticulate Fe-(oxyhydr)­oxides^52^ that promote As immobilization, elevated As and PAH concentrations may suppress these taxa, shifting Fe cycling toward alternative, partially abiotic or chemically mediated, pathways under electrochemical stimulation.

The microbial community data further suggest a limited biological transformation of As into more mobile forms. The low abundance of methylated As species (e.g., DMA) indicates minimal microbial methylation activity, which is supported by the relatively low representation of taxa known to actively methylate As. This reduces the likelihood of forming more mobile organoarsenic species and supports the potentially long-term stability of Fe-mediated As immobilization under current conditions. Nevertheless, interactions between PAH and As complicate this stabilization framework. At GW3 and GW4, elevated concentrations of PAH and As coincided with microbial communities enriched in PAH-degrading and As-tolerant taxa, such as *Pseudomonas*, *Rugosibacter*, *Duganella*, and *Acidovorax*. While these organisms may contribute positively to contaminant transformation, the accumulation of dissolved organic carbon (DOC) and PAH degradation intermediates can also enhance the As mobility. Organic ligands can complex Fe, reducing Fe–As coprecipitation efficiency, and can compete with arsenate for sorption sites on Fe-oxyhydroxides.^64^ Similar competitive and complexation effects have been shown to increase As mobilization in organic-rich soils.^65^

These observations suggest that cocontaminated systems containing both PAH and As may benefit from a sequential remediation strategy. Prioritizing PAH degradation, potentially through electricity-induced biostimulation of PAH-degrading microbial communities, could reduce organic competition for Fe binding sites and enhance subsequent As immobilization. This approach can be supported by the enrichment of known PAH-degrading bacteria and fungi at contaminated sites, indicating that the microbial community is responsive to both the contaminant availability and electrochemical stimulation. However, the effectiveness of Fe-based immobilization strategies may be constrained by additional geochemical factors. The presence of competing anions such as phosphate, silicate, and calcium, as well as nutrient amendments potentially introduced to stimulate microbial activity, can inhibit arsenate sorption and reduce Fe amendment efficiency.^66^ These interactions highlight the need for careful balancing of biostimulation and geochemical stabilization processes, particularly in complex groundwater systems.

From a mechanistic perspective, the field pilot can be conceptualised as a coupled electrochemical–biogeochemical reaction system in which electrochemical processes act as the primary driver of remediation. Electrochemical oxidation continuously supplies reactive Fe, O_2_ and maintains oxidizing conditions, thereby enabling rapid formation of Fe-(oxyhydr)­oxides and initial As immobilization. Microbial processes play a secondary but important role, primarily by codegrading organic contaminants that would otherwise inhibit sorption and by influencing Fe redox cycling at the microscale.^67^

In summary, under favorable conditions, such as water-saturated sandy soils with stable oxic conditions, the electricity-induced degradation and stabilization approach tested here demonstrated considerable potential for scalable, low-energy remediation. The combined action of electrochemical Fe cycling, microbial Fe oxidation, and biologically driven contaminant transformation offers a promising integrated strategy, particularly for legacy industrial or wood-treatment sites where multiple contaminant classes coexist. Importantly, the observed microbial and fungal community responses suggest that such systems can self-reinforce contaminant degradation and stabilization processes when appropriate redox and pH conditions are maintained.

The technology can be readily extended to beyond the experimental site. Both the system geometry and the grid layout can be adapted to site-specific conditions including soil heterogeneity and contamination depth. The applied electrode spacing (5 m) supports the practical scalability of the method. Moreover, the low electrical current requirements (Table S3) mean that energy consumption is not a dominant cost factor. Calculations of energy use and associated CO_2_ emissions over the experimental period are provided in the Supporting Information.

## 4 Conclusions

This two-year pilot study demonstrates that low-voltage electricity-stimulated groundwater treatment can effectively address mixed contamination by PAH and As at former wood-treatment sites. Across all monitoring wells, dissolved PAH concentrations decreased substantially, by 62–94% compared to initial levels, with simultaneous reductions across low-, medium-, and high molecular weight fractions, indicating that the treatment promoted oxidation beyond the lighter PAH species typically degraded during bioremediation. Importantly, oxygenated and nitrogen-containing PAH transformation products did not accumulate in groundwater, suggesting that secondary pollution risks associated with intermediate oxidation products remained minimal.

Dissolved As concentrations also generally declined with the clearest response observed in wells with comparatively low PAH levels. In more heavily contaminated and peat-rich zones, a decrease in dissolved As concentrations was weaker and more variable, indicating that high organic matter content and elevated PAH concentrations may inhibit Fe–As interactions. Nevertheless, arsenate remained the dominant As species (>95% in most samples), and reducing conditions that could promote Fe dissolution and As remobilization were not observed, indicating that the method did not induce adverse changes to As speciation or mobility.

Microbial community analyses revealed notable shifts associated with contamination gradients but no indication of treatment-related ecological disruption. Several bacterial genera associated with PAH degradation or As metabolism increased in relative abundance at more contaminated wells, whereas fungal communities showed a little systematic variation.

Overall, the findings indicate that electricity-induced spreading of Fe, combined with electrochemical oxidation, can simultaneously promote remediation of As- and PAH-contaminated groundwater with limited secondary effects. The approach shows promise for scaling up at complex, cocontaminated sites, although remediation efficiency may depend on local soil characteristics, particularly organic matter content and contaminant distribution. Since a decrease in dissolved As was more pronounced at low PAH concentrations, promoting the rapid degradation of PAH is essential to accelerate As immobilization, ensuring more effective remediation of cocontaminated soil.

## Supplementary Material



## Notes

Methods and Supporting Results of soil properties, concentrations of PAH degradation products, consumption of electricity and CO_2_ release during the experiment, speciation of As in groundwater, relative As bioavailability in soil, as well as prokaryotic family community and bioremediating fungal genera compositions and associations with As and PAH16 contamination (PDF)
