# Supplementary material for: Electricity-Induced Simultaneous in Situ Remediation of Arsenic and Polycyclic Aromatic Hydrocarbons in Groundwater at a Former Wood Treatment Site – a Field Pilot Study
Source: ACS ES T Water. 2026 Jun 2;6(6):3922–37. doi: 10.1021/acsestwater.6c00318 (PMC13274490; doi:10.1021/acsestwater.6c00318)
Supplement: Supplementary file 1 [file ew6c00318_si_001.pdf]

SUPPORTING INFORMATION

Electricity-induced simultaneous *in situ* remediation of arsenic and polycyclic aromatic hydrocarbons in groundwater at a former wood treatment site – a field pilot study

Jurate Kumpiene [a]*, Ivan Carabante[a], Erkki Lindberg[b], Spencer Long[c], Nicola Pratt[c], Phyllis Lam[c], Andrew Cundy[c]

[a] Waste Science and Technology, Luleå University of Technology, 97187 Luleå, Sweden

[b] Ekogrid Oy, Nuijamiestentie 7, 00400 Helsinki, Finland

[c] School of Ocean and Earth Science, University of Southampton, National Oceanography Centre Southampton, European Way, Southampton SO14 3ZH, Hampshire, UK

*Email: jurate.kumpiene@ltu.se

Contents

S1. METHODS 2

S1.1. EKOGRID™ Technology Description 2

S1.2. Soil sampling and analyses 3

S1.3. Arsenic bioavailability in soil 3

S1.4. Calculations of energy use and associated CO_2 emissions over the experimental period 3

S2. RESULTS 5

Table S1 5

Table S2 6

Table S3 8

Figure S1 9

Figure S2 9

Figure S3 11

Figure S4 11

Figure S5 12

Figure S6 13

Figure S7 133

Figure S8 15
S3. REFERENCES 16

S1. METHODS

S1.1. EKOGRID™ Technology Description

The electrokinetic treatment system used in this study - EKOGRID™ - is based on the application of low-voltage pulsed direct current to porous media. The system was originally developed for moisture control in masonry and fine-grained mineral soils but has subsequently been applied in environmental remediation contexts.

The technology operates using short, constant-voltage pulses with alternating polarity. Output voltage typically ranges between 7 and 16 V. In the standard pulsed configuration, successive voltage pulses are applied with reversed polarity. Pulse duration and sequence can be programmed via a dedicated control unit, allowing adjustment of operational mode depending on treatment objectives.

S1.1.1. Electrokinetic Mechanisms

Under an applied electric field, transport processes in porous media include electroosmosis and electromigration. Electroosmosis refers to the movement of pore water associated with hydrated cations within the electrical double layer at negatively charged mineral surfaces. Electromigration contributes to the transport of dissolved ionic species. Together, these mechanisms enable movement of pore water and dissolved constituents through fine-grained mineral soils.

According to technical information provided by the manufacturer, pulsed polarity reversal promotes repeated disturbance of the electrical double layer, potentially enhancing electroosmotic flow compared to continuous direct current. The applied electric field may also induce electrochemical reactions in the pore water, resulting in measurable changes in physicochemical parameters such as dissolved oxygen (DO), oxidation–reduction potential (ORP), and pH.

S1.1.2. Operational Modes

Two principal operational modes were available in the system used in this study:

(1) *Symmetric pulsed mode (electrochemical oxidation mode).* In this configuration, pulses of equal duration (typically 40–100 ms) are applied in alternating polarity. This mode is intended to promote distributed electrochemical reactions within the treated soil volume while limiting net directional water transport. Under these conditions, limited corrosion of steel electrodes is reported, resulting in minimal release of ferrous ions to the soil matrix.

(2) *Asymmetric pulsed mode (directional electroosmotic transport mode).* In this configuration, a longer pulse is applied in one polarity (e.g., up to 2000 ms) and a shorter reversed pulse (e.g., ~50 ms) in the opposite direction. This generates net electroosmotic flow from anode to cathode and can be used to enhance the distribution of injected amendments or irrigation water. Increased electrode corrosion may occur in this mode, leading to the release of ferrous ions into the pore water.

S1.1.3. Soil Requirements

Effective electrokinetic treatment requires sufficient soil moisture to ensure ionic conductivity; full saturation is not necessary. The method is most effective in mineral soils with negatively charged particle surfaces and measurable zeta potential (e.g., clays and silts). High organic matter content may reduce electrokinetic efficiency.

S1.2. Soil sampling and analyses

Soil samples were collected during the installation of groundwater wells and at the end of the experiment in the vicinity of the wells using a drilling rig. A fraction of the samples was sent for PAH analyses to Eurofins, Sweden, and the other fraction was taken to the LTU laboratory for analysis of pH, EC, organic matter content, As and Fe concentrations and As bioavailability. The As and Fe concentrations were measured in air-dried soil samples using a X-ray fluorescence analyser (Niton XL3t, Thermo Fisher Scientific).

S1.3. Arsenic bioavailability in soil

The air-dried soil samples were sieved to <0.250 mm and subjected to an *in vitro* assay that was performed to simulate the human gastric phase to quantify the bioaccessible fraction during direct soil ingestion by humans before and after the field experiment.[1] Briefly, 0.5 g soil was shaken (30 rpm) with 50 mL 0.4 M glycine (pH 1.5) for 2 h at 37 °C in a water bath. The samples were filtered through 0.45 µm nitrocellulose membrane filters and analysed for As using ICP-OES (PerkinElmer Optima 8300). *In vivo* relative As bioavailability was calculated as follows: *In vivo* relative As bioavailability (%) = 1.656 + (0.992) *in vitro* bioaccessible fraction in simulated gastric phase (%).[1]

S1.4. Calculations of energy use and associated CO_2 emissions over the experimental period

The electrical input to the site was monitored using an electricity meter, allowing precise monthly measurement of total energy consumption over the duration of the experiment. The measured values were used to calculate carbon dioxide (CO_2) release.

These CO_2 emissions associated with electricity use during the treatment of the site were estimated and compared with an alternative remediation scenario involving excavation and off-site disposal. In the latter case, only emissions from fuel consumption during transportation of

contaminated soil from the site and clean backfill material to the site were considered, while emissions related to excavation, soil handling, and landfill operations were not included.

The following assumptions were made:

- a medium-duty truck (~15–16 tonnes payload) typically consumes approximately 25 L per 100 km (≈0.25 L/km); [2]
- diesel combustion emits about 2.6–2.7 kg CO_2 per litre of fuel, corresponding to approximately 0.65–0.68 kg CO_2 per km;[3]
- Electricity production in Sweden has historically averaged approximately 20 g CO_2/kWh.[4]

S2. RESULTS

Table S1. pH, electrical conductivity (EC) and organic matter (OM) content in soil at various depths of sampling points.

Sampling point	Depth (m)	pH	EC (µS/cm)	Redox (mV)	OM (%)*	Soil type
GW Ref1	0 - 0.6	5.99	25.4	226	0.7	sand
	0.6 - 0.65	5.81	143.3	200		sand/slag
	0.65 - 1	5.60	74.1	178	67.8	peat
	1 - 1.5	5.96	58.1	172	64.5	peat
	1.5 - 2	4.88	70.5	203		
	2 - 2.2	5.12	197.1	178		
GW 1	0 - 0.1	5.59	42.9	266	8.3	sand
	0.1 - 0.35	6.19	3.7	219		
	0.35 - 0.5	5.75	25.2	407		
	0.5 - 0.8	5.22	39.2	418		
	0.8 - 1	4.56	65.5	408	43.3	peat
	1 - 1.5	5.11	83.7	252	21.3	peat/sand mix
	1.5 - 1.8	5.35	48.9	208		
	1.8 - 2.2	4.47	83.9	331		
GW 2	0 - 0.4	5.98	66.0	295	40.6	peat
	0.4 - 0.5	5.52	69.3	316		
	0.5 - 1	5.09	111.0	330	87.8	peat
	1 - 1.5	5.30	52.5	238	71.0	peat
	1.5 - 2.2	5.34	38.24	139	29.0	peat/sand mix
GW 3	0 - 0.1	5.75	7.4	236	2.7	sand
	0.1 - 0.45	6.39	6.4	223		
	0.45 - 0.6	7.78	80.2	203		
	0.6 - 1	6.13	27.9	155	64.3	peat
	1 - 1.5	5.60	49.0	198	30.9	peat/sand mix
	1.5 - 2.2	5.98	34.3	81	0.5	sand
GW 4	0 - 0.5	6.12	14.2	159	2.2	sand
	0.5 - 0.7	6.76	7.3	199	13.7	slag
	0.7 - 1	6.36	79.7	110	56.3	peat
	1 - 1.5	5.71	38.2	157		
	1.5 - 2.2	5.81	17.9	145		
GW 5	0 - 0.6	6.06	47.9	155	1.0	sand
	0.6 - 1	5.97	50.6	189	42.7	peat/sand mix
	1 - 1.1	6.08	24.7	197	4.1	sand
	1.1 - 1.8	5.86	36.2	156		
	1.8 - 2.2	5.50	73.1	152	16.6	sand

* Measured as loss on ignition at 550 °C.

Table S2. Concentrations of oxygenated PAH (oxy-PAH) and nitrogen-containing PAH (N-PAH) compounds in groundwater at the experimental site analysed on five occasions during the second year of the experiment. < - values below method detection limits.

	Compound	Sampling point				
September, 2023		GVRef1	GV1	GV2	GV3	GV4
Oxy-PAH	1,9-Benz-10-anthrone	<0.01	<0.01	<0.01	<0.10	0.072
	1-Indanone	<0.1	<0.10	<0.10	2.1	1.1
	2-Methylanthraquinone	<0.01	<0.01	<0.01	0.097	0.049
	4H-Cyclopenta(def)phenanthren-4-one	<0.01	<0.01	0.015	0.51	0.057
	5,12-Naphthacenequinone	<0.01	<0.01	<0.01	0.044	0.058
	6H-Benz(cd)pyrene-6-one	<0.01	<0.01	<0.01	<0.01	<0.01
	7,12-Benzo(a)anthracenequinone	<0.01	<0.01	<0.01	<0.10	<0.10
	9,10-Anthraquinone	<0.01	0.016	0.056	0.74	0.2
	9-Fluorenone	<0.01	0.014	0.12	1.1	0.19
	Benzo(a)fluorenone	<0.01	<0.01	<0.01	0.026	0.076
N-PAH	Acridine	<0.1	<0.1	<0.2	1.5	1.6
	Carbazole	<0.1	<0.1	1.1	14	6.9
	Quinoline	<0.1	<0.1	<0.1	<0.1	0.43
December, 2023		GVRef1	GV1	GV2	GV3	GV4
Oxy-PAH	1,9-Benz-10-anthrone	<0.05	<0.05	<0.05	<0.05	<0.05
	1-Indanone	<0.05	<0.05	<0.05	0.17	1.9
	2-Methylanthraquinone	<0.05	<0.05	<0.05	<0.05	<0.05
	4H-Cyclopenta(def)phenanthren-4-one	<0.05	<0.05	<0.05	0.28	<0.05
	5,12-Naphthacenequinone	<0.05	<0.05	<0.05	<0.05	<0.05
	6H-Benz(cd)pyrene-6-one	<0.05	<0.05	<0.05	<0.05	<0.05
	7,12-Benzo(a)anthracenequinone	<0.05	<0.05	<0.05	<0.05	<0.05
	9,10-Anthraquinone	<0.05	<0.05	<0.05	0.2	0.12
	9-Fluorenone	<0.20	<0.20	<0.20	<0.50	<0.20
	Benzo(a)fluorenone	<0.05	<0.05	<0.05	<0.05	<0.05
N-PAH	Acridine	<0.2	<0.2	<0.2	1.6	4.5
	Carbazole	<0.05	<0.05	0.29	4.9	3.1
	Quinoline	<0.2	<0.2	<0.2	<0.2	<0.2
February, 2024		GVRef1	GV1	GV2	GV3	GV4
Oxy-PAH	1,9-Benz-10-anthrone	<0.01	<0.01	<0.01	0.019	0.027
	1-Indanone	<0.10	<0.10	<0.10	5.1	0.46
	2-Methylanthraquinone	<0.01	<0.01	<0.01	0.066	0.069
	4H-Cyclopenta(def)phenanthren-4-one	<0.01	<0.01	0.16	1.3	0.4
	5,12-Naphthacenequinone	<0.01	<0.01	0.021	0.021	0.032
	6H-Benz(cd)pyrene-6-one	<0.01	<0.01	<0.01	<0.01	<0.10
	7,12-Benzo(a)anthracenequinone	<0.01	<0.01	<0.01	<0.01	<0.10

	9,10-Anthraquinone	<0.01	<0.01	0.086	0.34	0.4
	9-Fluorenone	<0.01	<0.01	0.12	13	<0.10
	Benzo(a)fluorenone	<0.01	<0.01	0.027	0.1	<0.10
N-PAH	Acridine	<0.1	<0.1	0.17	0.83	0.19
	Carbazole	<0.1	<0.1	<0.1	<0.1	<1.0
	Quinoline	<0.1	<0.1	<0.1	<0.1	<1.0

May, 2024		**GVRef1**	**GV1**	**GV2**	**GV3**	**GV4**
Oxy-PAH	1,9-Benz-10-anthrone	<0.01	<0.01	<0.01	0.058	<0.01
	1-Indanone	<0.10	<0.10	<0.10	2.3	3.2
	2-Methylanthraquinone	<0.01	<0.01	<0.01	0.075	0.051
	4H-Cyclopenta(def)phenanthren-4-one	<0.01	<0.01	0.024	0.23	0.037
	5,12-Naphthacenequinone	<0.01	<0.01	<0.01	0.074	0.038
	6H-Benz(cd)pyrene-6-one	<0.01	<0.01	<0.01	<0.01	<0.01
	7,12-Benzo(a)anthracenequinone	<0.01	<0.01	<0.01	<0.05	<0.02
	9,10-Anthraquinone	<0.01	<0.01	0.015	0.29	0.38
	9-Fluorenone	<0.01	<0.01	<0.05	<0.10	<0.10
	Benzo(a)fluorenone	<0.01	<0.01	<0.01	0.052	<0.01
N-PAH	Acridine	<0.1	<0.1	<0.1	5.7	6.9
	Carbazole	<0.1	<0.1	0.71	11	28
	Quinoline	<0.1	<0.1	<0.1	<0.1	0.48

August, 2024		**GVRef1**	**GV1**	**GV2**	**GV3**	**GV4**
Oxy-PAH	1,9-Benz-10-anthrone	<0.01	<0.01	<0.01	0.012	1.3
	1-Indanone	<0.10	<0.10	0.13	13	9.7
	2-Methylanthraquinone	<0.01	<0.01	<0.01	0.058	<0.01
	4H-Cyclopenta(def)phenanthren-4-one	<0.01	<0.01	<0.01	1	<0.20
	5,12-Naphthacenequinone	<0.01	<0.01	<0.01	0.028	2.1
	6H-Benz(cd)pyrene-6-one	<0.01	<0.01	<0.01	<0.01	0.21
	7,12-Benzo(a)anthracenequinone	<0.01	<0.01	<0.01	<0.01	<0.10
	9,10-Anthraquinone	<0.01	0.012	0.011	0.38	<1.0
	9-Fluorenone	<0.01	<0.01	0.03	1.1	<0.10
	Benzo(a)fluorenone	<0.01	<0.01	<0.01	0.023	0.8
N-PAH	Acridine	<0.1	<0.1	<0.1	2	56
	Carbazole	<0.1	<0.1	0.17	5	<0.5
	Quinoline	<0.1	<0.1	<0.1	<0.01	9.9

Table S3. Consumption of electricity over the duration of the experiment in kWh.

Month \ Year	2022	2023	2024
January		28	33
February		27	30
March		32	32
April		31	31
May		31	32
June		30	32
July	29	30	34
August	28	34	22
September	28	37	
October	28	35	
November	28	36	
December	28	35	
Total per year	**169**	**386**	**246**

The calculated average energy consumption was 30 kWh per month to treat 450 m² contaminated site. Based on a total electricity consumption of approximately 800 kWh during the experimental period, the associated emissions amount to about 16 kg CO_2.

For comparison, a conventional excavation scenario was considered, where contaminated soil is transported to the nearest suitable treatment facility, located within a 100 km radius. The experimental site comprises approximately 1,500 tonnes of soil (450 m² × 2 m depth = 900 m³; assuming a bulk density of 1.66 t/m³).

Transporting this volume of soil using a medium-duty truck, along with an equivalent amount of clean backfill material, would require approximately 100 round trips (accounting for both removal of contaminated soil and delivery of clean soil), corresponding to a total driving distance of 20,000 km. This results in estimated CO_2 emissions of approximately 13,000 kg.

Even under a conservative assumption of continued operation over an additional 10 years, with an estimated electricity consumption of approximately 400 kWh per year (i.e. 4,000 kWh in total), the associated emissions would increase by only about 80 kg CO_2.

Overall, the in situ treatment approach results in CO_2 emissions that are several orders of magnitude lower than those associated with conventional excavation and transport-based remediation.

FIGURES

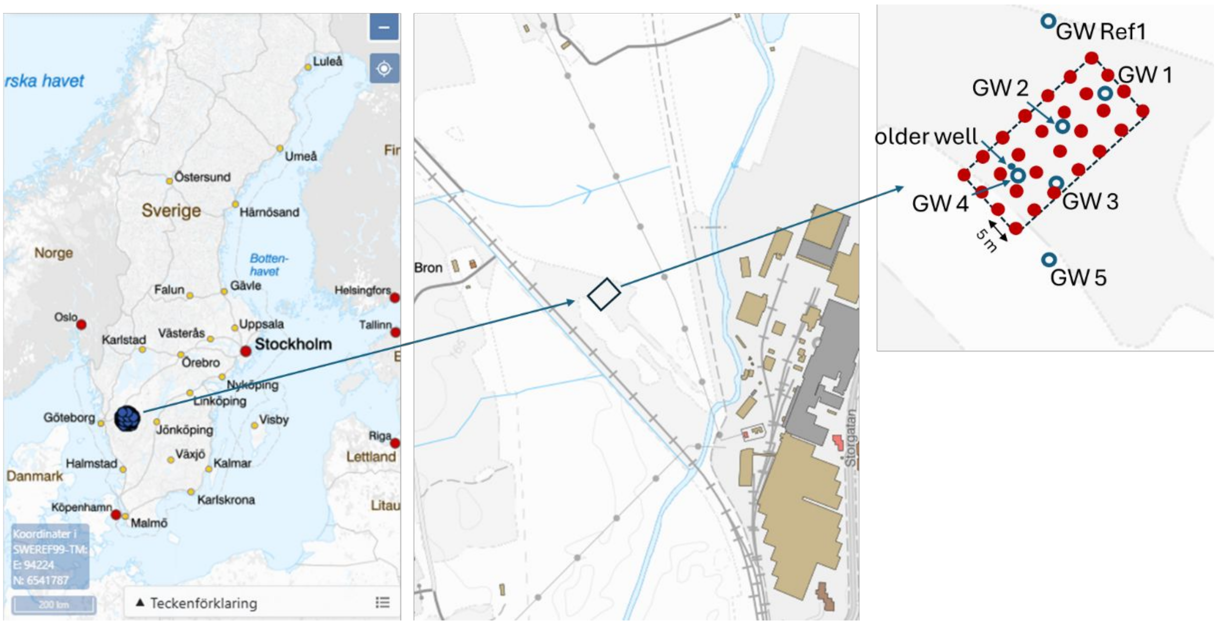

Figure S1. Location of the experimental site, schematics of the electrode grid (filled circles) and groundwater wells (hollow circles). Source of map: the Swedish Geological Survey's map viewer https://www.sgu.se/produkter-och-tjanster/kartor/kartvisaren/

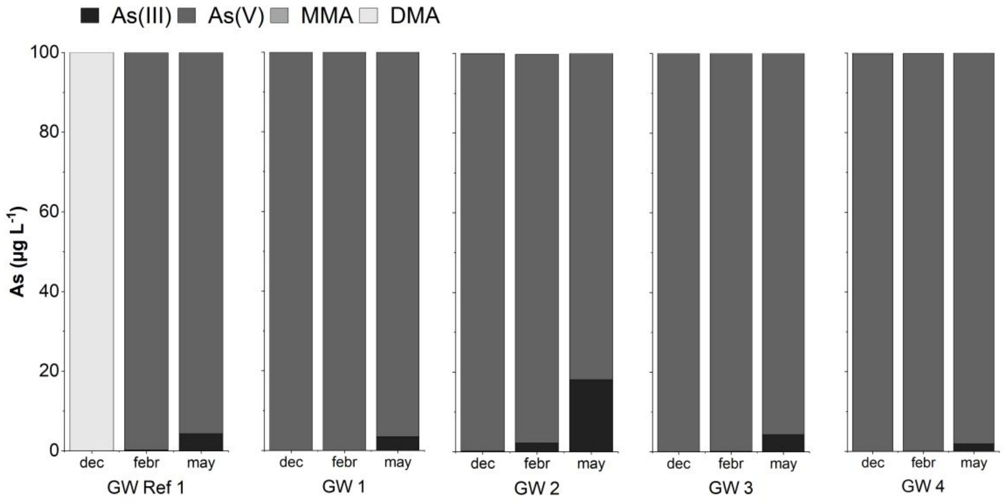

Figure S2. Speciation of As in groundwater measured on three occasions during the second year of the experiment. As(III) - arsenite; As(V) – arsenate; MMA – monomethylarsonate; DMA – dimethylarsinate.

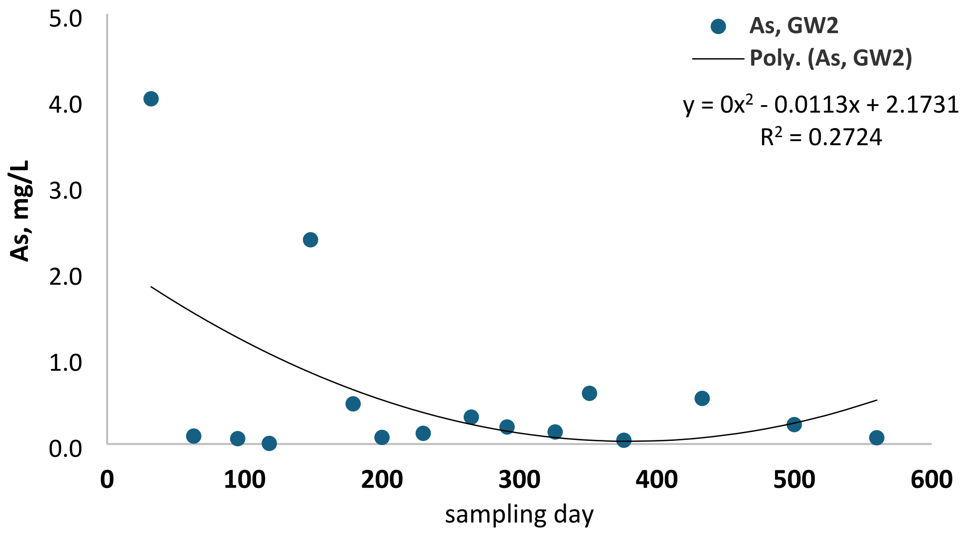
As, GW2
Poly. (As, GW2)
y = 0x2 - 0.0113x + 2.1731
R2 = 0.2724
As, mg/L
5.0
4.0
3.0
2.0
1.0
0.0
0
100
200
300
400
500
600
sampling day

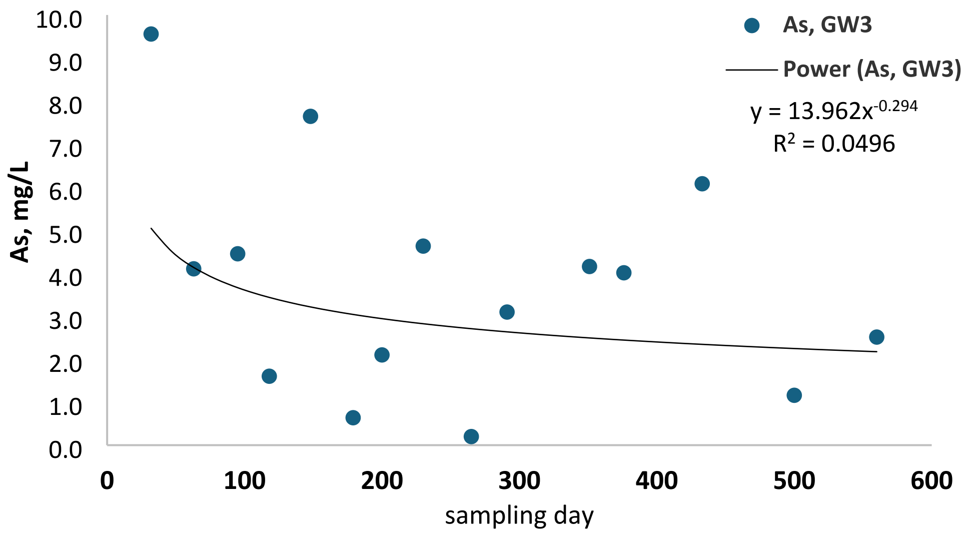
As, GW3
Power (As, GW3)
y = 13.962x-0.294
R2 = 0.0496
As, mg/L
10.0
9.0
8.0
7.0
6.0
5.0
4.0
3.0
2.0
1.0
0.0
0
100
200
300
400
500
600
sampling day

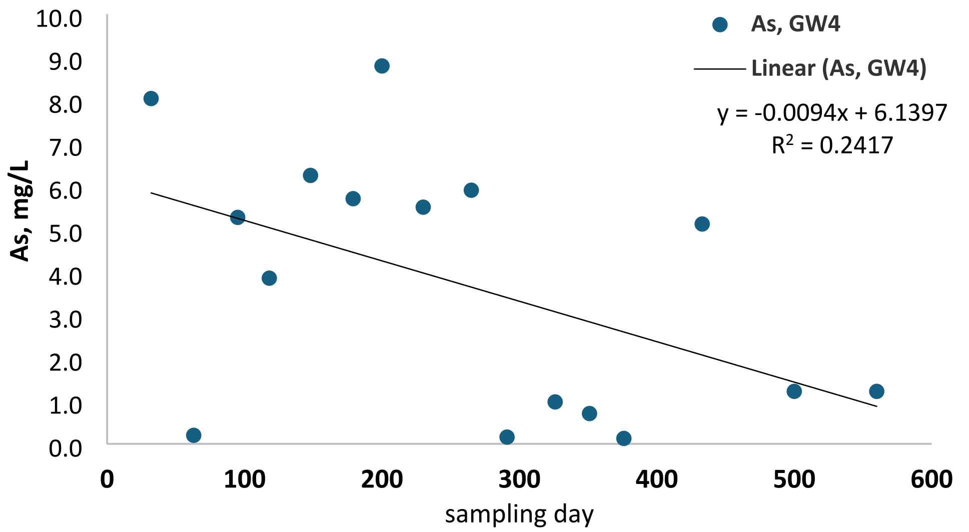
As, GW4
Linear (As, GW4)
y = -0.0094x + 6.1397
R2 = 0.2417
As, mg/L
10.0
9.0
8.0
7.0
6.0
5.0
4.0
3.0
2.0
1.0
0.0
0
100
200
300
400
500
600
sampling day

Figure S3. Fitted models of variations in As concentrations in groundwater of the wells GW2, GW3 and GW4. Values in GW Ref1 and GW1 were very low or below detection limits most of the time and thus not shown here.

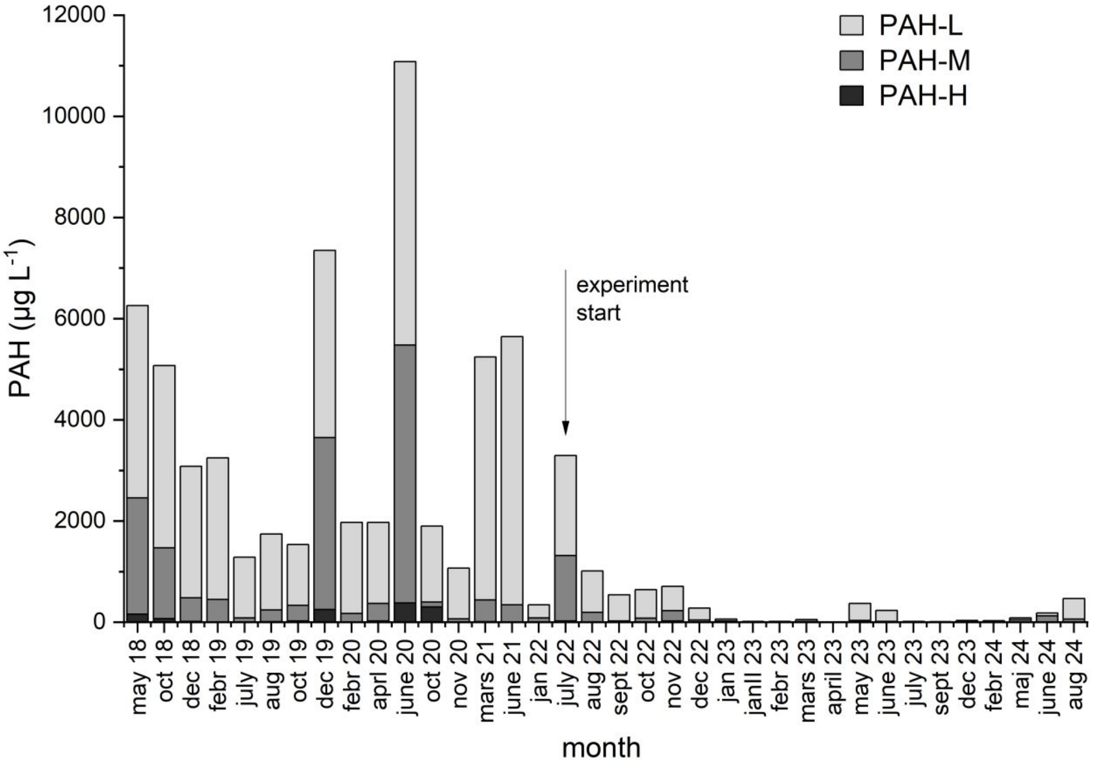

Figure S4. Concentration of PAH16 as a sum of low- (PAH-L), medium- (PAH-M) and high molecular weight PAH (PAH-H) in an older groundwater well at the pilot site in the vicinity to WG4 during four years prior to the experiment and those in GW4 during the two years of the experiment.

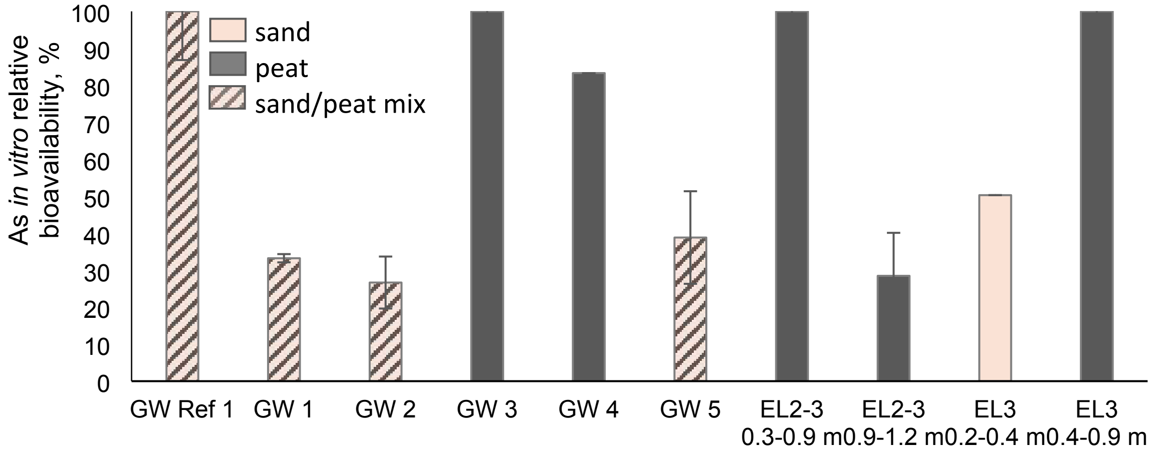

Figure S5. Estimated *in vivo* relative As bioavailability in soil at the end of the experiment at various depths. Additional sampling points: EL2-3 – by the electrode located between GW2 and GW3; EL3 – by the electrode located at GW3.

Analysis of *in vitro* relative As bioavailability was conducted across several soil types and depths at selected sampling points. The selected samples correspond to the layers with the highest As concentrations in the respective soil profile. The results showed a quite clear trend related to soil type. Namely, peat generally exhibited the highest As bioavailability, with values approaching 100% (Fig. S5). In contrast, sandy soil and sand/peat mixtures (representing transition zones) showed moderate bioavailability levels (25-40%), indicating lower extractability of As in these mixtures, likely due to the presence of Fe-As compounds. An exception was observed at the reference point, where the mixed soil contained close-to-background As concentration (13 mg kg^{-1}), but the entire amount was bioavailable. While sandy soils are typically characterized by low sorption capacity and generally higher As bioavailability compared to clay-rich soils[5], these results indicate that sandy soil conditions in the studied site were more favourable for As sorption compared to peat soil. These findings are consistent with As behaviour observed in the groundwater, where high soil organic matter content appeared to hinder sorption of As to Fe oxides, thereby maintaining elevated bioavailability in the peat-rich environment. From the risk management perspective, this means that sandy zones may require less *in situ* remediation efforts compared with peat-rich zones.

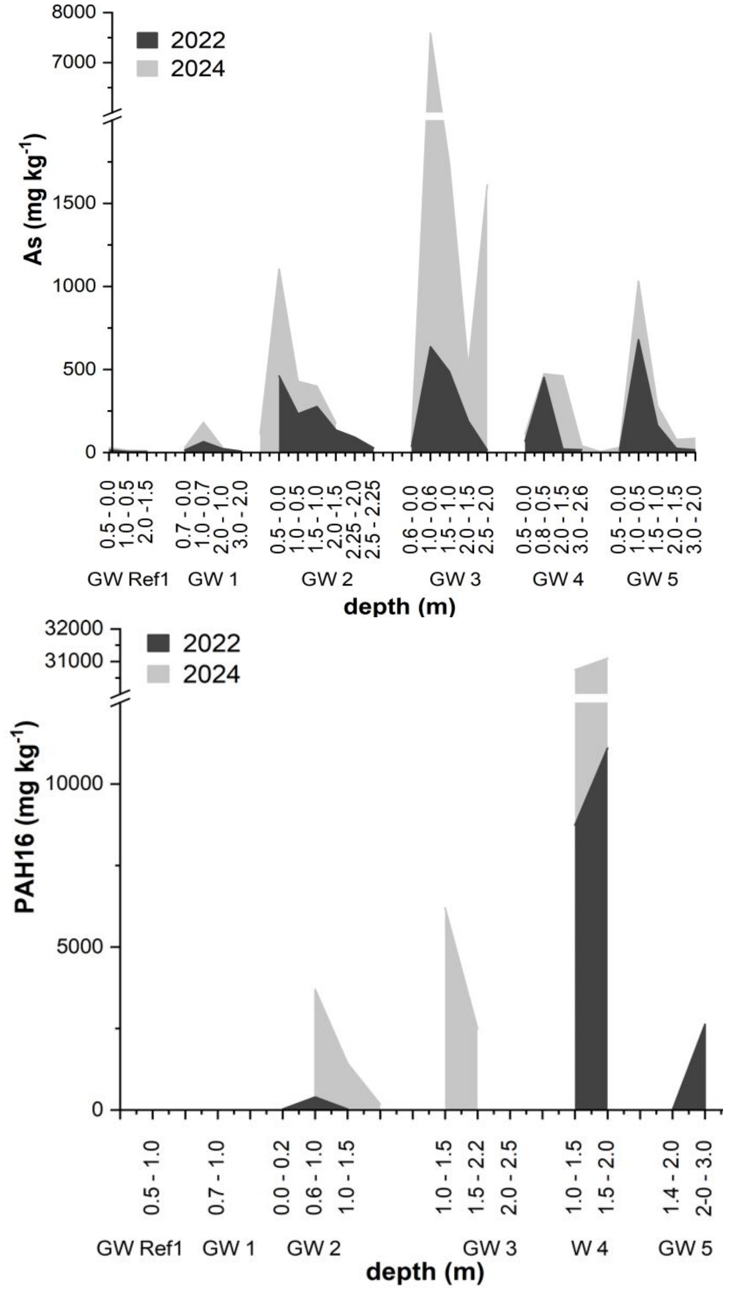

Figure S6. Total As and PAH concentrations in soil at various depths of the groundwater well locations measured during the installation of groundwater wells in 2022 and adjacent to those at the end of the experiment in 2024.

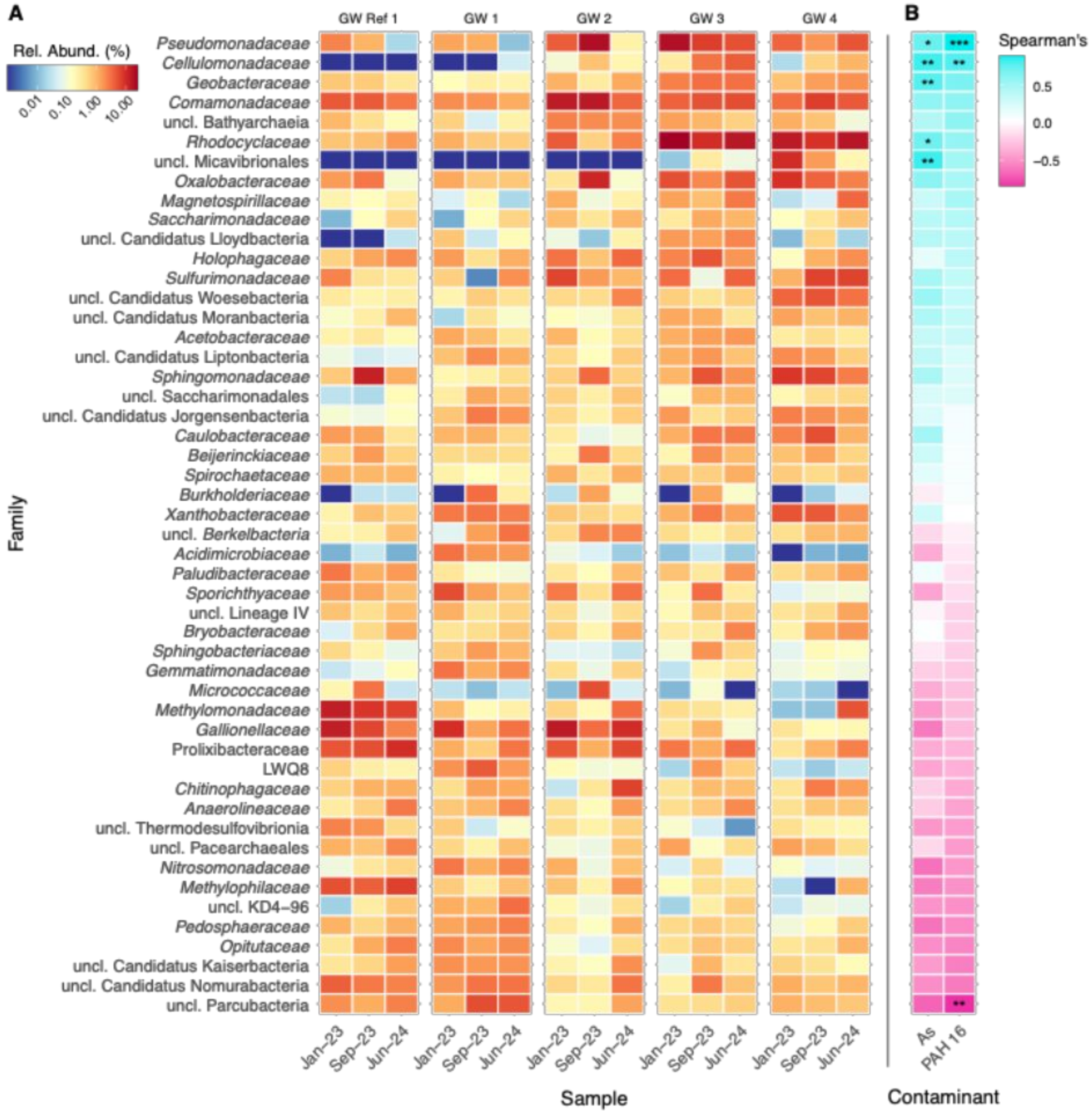

Figure S7. Prokaryotic family community compositions and associations with As and PAH16 contamination across groundwater samples. A: Heatmap showing the relative abundance (%) of key families across samples. B: Heatmap showing key family relative abundance Spearman's rank correlations with As and PAH16 concentrations. Families are sorted by their correlation with PAH16, with those showing the strongest positive associations placed at the top of the plot. Only the top 50 most relatively abundant families across samples are shown. Taxa preceded with uncl. could not be classified to the family level and hence are assigned to the lowest possible taxonomic rank.

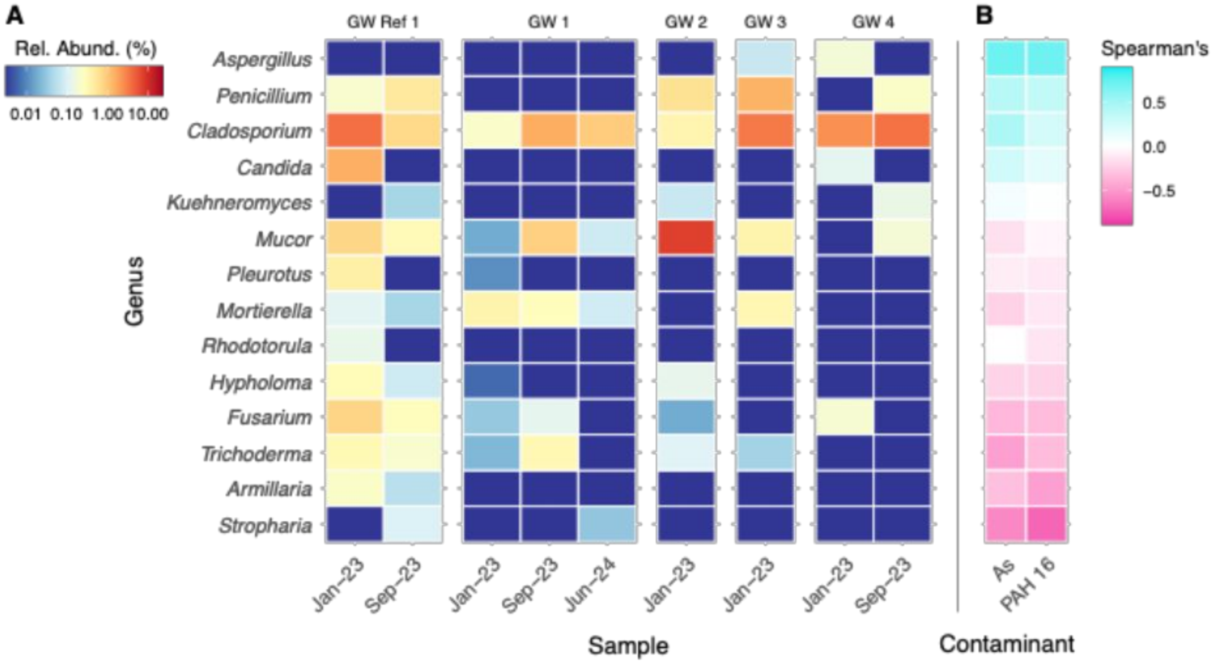

Figure S8. **Community compositions and associations with As and PAH16 contamination of known bioremediating fungal genera across groundwater samples.** A: Heatmap showing the relative abundance (%) of fungal genera with known bioremediating capabilities across samples. B: Heatmap showing corresponding genera relative abundance Spearman's rank correlations with As and PAH16 concentrations. Genera are sorted by their correlation with PAH16, with those showing the strongest positive associations placed at the top of the plot; note that none of the taxa shown exhibited significant correlations with As or PAH16, nor were significantly enriched in high (> 1 mg L^{-1}) contaminant samples. Fungal genera identified are reported earlier.[6-8]

S3. REFERENCES

(1) Juhasz, A.L., Weber, J., Smith, E., Naidu, R., Rees, M., Rofe, A., Kuchel, T., Sansom, L. 2009. Assessment of four commonly employed in vitro arsenic bioaccessibility assays for predicting in vivo relative arsenic bioavailability in contaminated soils. *Environmental Science and Technology,* 43, 9487–9494. DOI: 10.1021/es902427y

(2) Webfleet:https://www.webfleet.com/en_gb/webfleet/industries/transport/fuel-efficiency/?utm_

(3) Girteka:https://www.girtekagroup.com/eco-driving-in-europes-trucking-sector/?utm_source=chatgpt.com

(4) Nowtricity: https://www.nowtricity.com/

(5) Kumpiene, J.; Engström, K.; Pinedo Taquia, A.; Carabante, I.; Bjuhr, J. Arsenic immobilisation in soil using electricity-induced spreading of iron in situ. *J. Environ. Manage.* 2023, 325, Part A, 116467. DOI: 10.1016/j.jenvman.2022.116467

(6) Kadri T, Rouissi T, Kaur Brar S, Cledon M, Sarma S, Verma M. 2017. Biodegradation of polycyclic aromatic hydrocarbons (PAHs) by fungal enzymes: A review. *Journal of Environmental Science – China,* 51, 52-74. doi: 10.1016/j.jes.2016.08.023.

(7) Patel AB, Shaikh S, Jain KR, Desai C, Madamwar D. 2020. Polycyclic Aromatic Hydrocarbons: Sources, Toxicity, and Remediation Approaches. *Frontiers in Microbiology,* 11, 562813. doi: 10.3389/fmicb.2020.562813.

(8) González-Benítez N, Durante-Rodríguez G, Kumar M, Carmona M. 2021. Editorial: Biotechnology for Arsenic Detection and Bioremediation. *Frontiers in Microbiology,* 12, 743109. doi: 10.3389/fmicb.2021.743109.
